# Linkage mapping and quantitative trait loci analysis of sweetness and other fruit quality traits in papaya

**DOI:** 10.1186/s12870-019-2043-0

**Published:** 2019-10-26

**Authors:** Usana Nantawan, Chutchamas Kanchana-udomkan, Ido Bar, Rebecca Ford

**Affiliations:** Environmental Futures Research Institute, School of Environment and Sciences, Griffith University, 170 Kessels Road Nathan, Nathan, QLD 4111 Australia

**Keywords:** Papaya breeding, Fruit quality trait, GBS, QTL analysis, SNP markers

## Abstract

**Background:**

The identification and characterisation of quantitative trait loci (QTL) is an important step towards identifying functional sequences underpinning important crop traits and for developing accurate markers for selective breeding strategies. In this study, a genotyping-by-sequencing (GBS) approach detected QTL conditioning desirable fruit quality traits in papaya.

**Results:**

For this, a linkage map was constructed comprising 219 single nucleotide polymorphism (SNP) loci across 10 linkage groups and covering 509 centiMorgan (cM). In total, 21 QTLs were identified for seven key fruit quality traits, including flesh sweetness, fruit weight, fruit length, fruit width skin freckle, flesh thickness and fruit firmness. Several QTL for flesh sweetness, fruit weight, length, width and firmness were stable across harvest years and individually explained up to 19.8% of the phenotypic variance of a particular trait. Where possible, candidate genes were proposed and explored further for their application to marker-assisted breeding.

**Conclusions:**

This study has extended knowledge on the inheritance and genetic control for key papaya physiological and fruit quality traits. Candidate genes together with associated SNP markers represent a valuable resource for the future of strategic selective breeding of elite Australian papaya cultivars.

## Background

Papaya (*Carica papaya* L.) is one of the top five produced tropical fruit crops, listed as a super fruit in the fight against vitamin deficiency [24, 51]. Global annual production of papaya is approximately 11.22 metric tons (Mt), increasing 4.35% per year [23]. In Australia, papaya is an important domestic fresh fruit crop with just 6.5 thousand tons grown annually [24]. The industry is currently relatively small but with large potential to expand to meet the growing global market demand.

Novel and advanced breeding tools will enable faster and more accurate selection of key consumer-driven traits. As such, marker-assisted selection (MAS) has been introduced in papaya breeding programs elsewhere to efficiently develop superior varieties with desired traits [6, 64, 66]. However, progress has been limited by a dearth of genomic information and few identified quantitative trait loci (QTL) associated with markers/sequences for trait selection.

Success in robust QTL identification is dependent on molecular marker map density, directly affecting map resolution, and accurate placement of qualitative data. Previous maps have varied in coverage and resolution. The ‘Sunrise Solo’ x Line UH356 map comprised 61 random amplified polymorphic DNA (RAPD markers distributed in 11 linkage groups (LG) over 999 cM. The subsequent ‘Kapoho’ x ‘Sunup’ map of Ma et al. [41] comprised 1498 amplified fragment length polymorphism (AFLP) loci in 12 LG over 3294 cM. Later, the ‘AU9’ x ‘Sunup’ map of Chen et al. [15] comprised 706 simple sequence repeat (SSR) markers in 12 LG over 1070 cM, within which elongated fruit shape was associated with a QTL in LG1. Blas et al. [5] then exploited the same mapping population and constructed a map comprising 712 SSR and 277 markers in 14 LG and over 945 cM. Meanwhile, the whole genome sequence of papaya ‘Sunup’ was released by Ming et al. ([46]; http://www.plantgdb.org/CpGDB), making the integration of physical and high-density genetic maps possible [79]. Due to narrow genetic base of papaya within the cultigen [52], a preliminary investigation on these SSR markers on our selected parental lines showed only 16.67% polymorphisms and predicted to cover only 120 loci (Unpublished data). Therefore, single nucleotide polymorphic (SNP) based mapping was introduced to speed-up and uncover the development of linkage maps and the identification of key genomic locations underlying complex traits, including flesh sweetness and other fruit quality traits in papaya.

Once aligned within the linkage map, the identification of putative candidate genes that underlie the major QTL and potentially contribute towards trait expression may be possible. Functionally validated markers may then represent sequences useful in selective breeding strategies. Previously in papaya, QTL for plant height, stem diameter and number of node at first flowering were mapped using RAPD markers in a population of 253 F_2_ plants (‘Sunrise Solo’ x Line UH356 [64];). From two to four QTLs were identified for each trait, which explained 42, 37 and 30% of the total phenotypic variance observed in plant height, stem diameter and number of node at first flowering, respectively. Blas et al. [6] subsequently identified 14 QTL controlling fruit weight, length, width and shape with phenotypic effects ranging from 5 to 23%. These were mapped on LG 2, 3, 7 and 9 using a population of 219 F_2_ ‘Khaek Dum’ x Line 2H94 plants.

The identification of reliable markers for selective breeding purposes that are associated with major QTL conditioning a trait of interest is reliant on the genetic stability of the markers with which the QTL has been associated. Indeed, through mutation and/or selective evolution, the sequences residing in close proximity to major QTL may vary among genetic backgrounds. Also, recombination events among different populations, even produced from the same parents, may not be conserved and hence marker transferability is not assured among genotypes or populations [33, 65]. Therefore, individual high-density genetic linkage maps are required for the identification of the genetic loci conditioning key fruit quality traits of a particular genotype.

High density maps are generated via a genotyping by sequencing (GBS) approach, for rapid and cost-effective high-throughput SNP marker discovery [21]. This approach has been applied for uncovering fruit quality trait QTL in zucchini [48] and tomato [11]. Both studies found GBS to be a highly efficient technology for QTL analysis and candidate gene mining. The construction of a genetic map of zucchini was performed using 120 F_8_ from an inter-subspecific cross between zucchini and scallop (ssp. *pepo* x ssp. *ovifera*). In total, 48 consistent QTL for vine, flowering and fruit quality traits were detected based on three environments analyses. These QTL were distributed across 33 independent positions across 15 LGs and each QTL explained from 1.5 to 62.9% of the phenotypic variance. Eight stable QTL related to leaf incision, fruit shape and length, and rind and flesh colour of zucchini were reported along with their underlying candidate genes. In tomato, Celik et al. [11] utilised a genetic map of 93 individuals from a backcross of *Solanum lycopersicum* ‘Tueza’ and *Solanum pimpinellifolium* (LA1589) for QTL mapping and selection of favourable alleles for 11 desired fruit quality traits. A total of 37 QTL affecting fruit quality of tomato were detected, explaining from 3 to 47% of the phenotypic variation. Among these, three were detected for fruit weight, nine for flesh colour, two for skin colour and four for each of fruit firmness, fruit shape and sugar content [11].

The advantages of GBS technology holds great promise for simplifying the construction of high-density maps and identifying QTL linked to quality fruit traits in papaya, which has a narrow genetic base and a low rate of sequence diversity [34, 55, 67, 72]. With the increase in information available in the sequence databases, GBS and candidate genes approaches can be combined to speed up the development of new markers for marker-assisted breeding programs [57].

This study focused on linkage mapping and QTL analysis for fruit quality traits in a papaya F_2_ population developed from the cross ‘RB2’ x ‘Sunrise Solo’. The aims were 1) to identify the locations of the major genetic components conditioning sweetness, fruit weight, fruit length, fruit width, skin freckle, flesh thickness and fruit firmness and 2) Identify and characterize the putative sweetness candidate genes to determine their potential for use in future marker-assisted selection strategies.

## Results

### Sequence data and SNP discovery

A total of 57.78 Gb of sequence data, comprising 577.7 million reads, was generated from the parents and 226 F_2_ samples. Following mapping to the ‘Sunup’ reference genome of Ming et al. [46], 44,030 SNPs were identified. After filtration to remove SNPs with more than 80% missing data and/or low read depth, 1701 high quality SNPs remained (3.86%). Subsequently, duplicated and monomorphic SNPs were excluded, resulting in 1302 (2.95%) for map construction with a density of 1 SNPs per 285.7 kb.

### Linkage map construction

Of the resultant sub-set of high quality 1302 SNPs, a total of 1153 were used to create the initial map of ‘RB2’ x ‘Sunrise Solo’ (Additional file 4: Table S3, Additional file 6: Figure S2). This comprised 23 LG, 15 major and 6 minor, spanning 3096.93 cM with an average marker interval of 2.7 cM. However, 882 (76.4%) of the markers were distorted in their expected segregation ratio (1:2:1) within the F_2_ population. Among these, 187 (21.3%) were skewed towards the female parent (‘RB2’) and 98 (11.2%) were skewed towards the male parent (‘Sunrise Solo’). The remaining 597 distorted markers were skewed towards an heterozygous genotype (Additional file 5: Table S4).

**Fig. 1:**
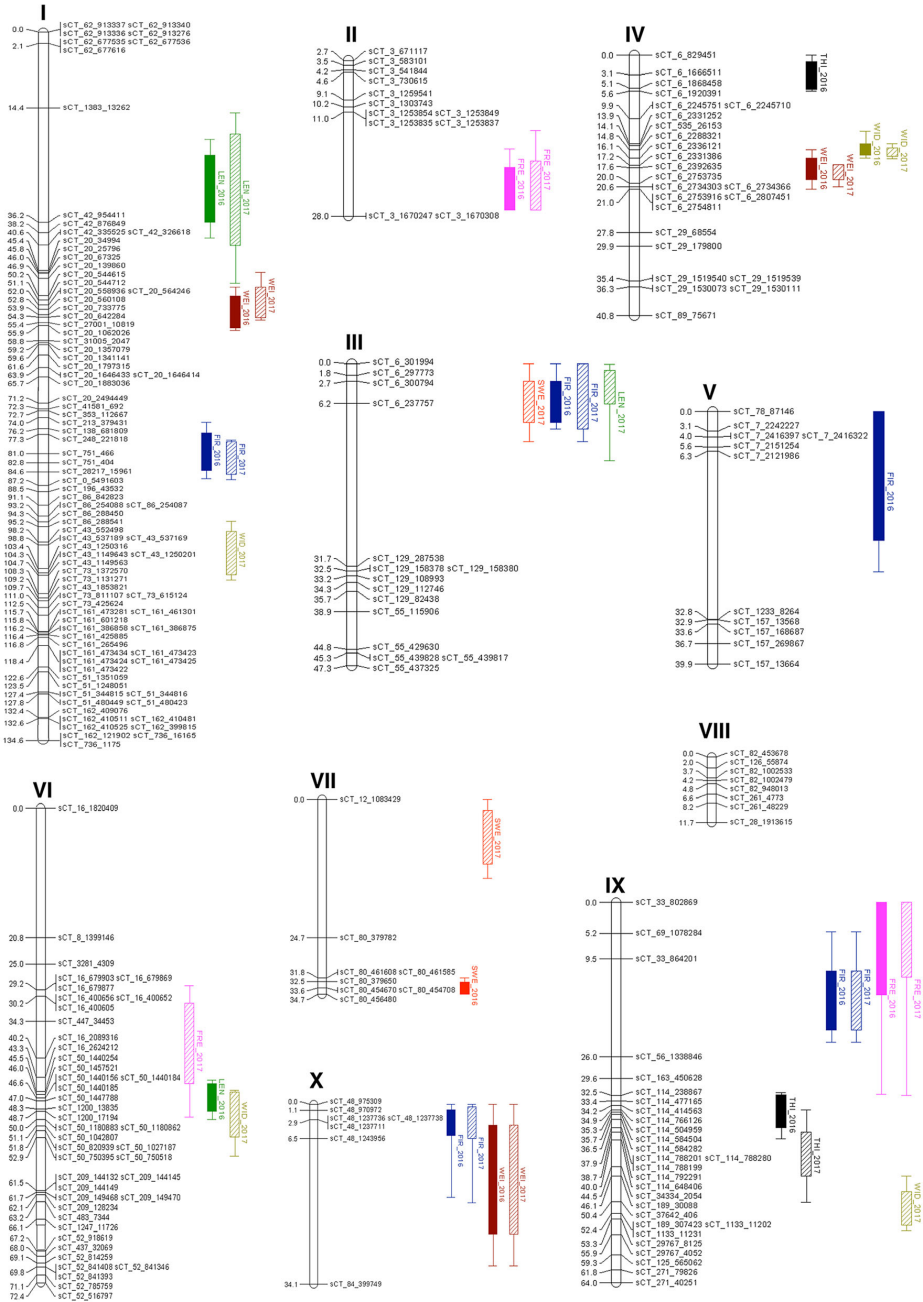
The genetic map of ‘RB2’ x ‘Sunrise Solo’ with QTL for fruit quality traits. The LG are labelled I-X. The left pane indicates the genetic map position in cM of each SNP. Colour bars on the right of the map indicate QTL position and logarithm of odds (LOD) interval at 95% confidence; where flesh sweetness (SWE) – red; fruit weight (WEI)-brown; fruit length (LEN)-green; fruit width (WID)-olive; skin freckle (FRE)-pink; flesh thickness (THI)-black; fruit firmness (FIR)- blue. Data from harvest year 2016 and 2017 are represented in solid and diagonal-stripe bar, respectively

Of the 1153 initial mapped SNP markers, only 271 segregated as expected (*p*-value ≥0.05) and following revision of the linkage analyses, 52 remained unlinked. Therefore, the final map consisted of 219 SNP loci within 10 LG (I to X; Table 1 and Fig. 1). Each LG comprised from 3 to 75 SNPs and ranged from 2.2 cM to 134.6 cM in length with average gaps between SNP of 3.5 to 27.6 cM. The final map spanned 509.7 cM, approximately six-times smaller than the initial map.

**Table 1:** Summary of the final linkage map of the F_2_ population (‘RB2’ x ‘Sunrise Solo’)

LG	No. of SNP loci	Length (cM)	Av. marker interval (cM)	Gap^a^(cM)	% SNP loci^b^
I	75	134.6	1.8	21.8	34.2
II	10	28	2.8	17	4.5
III	18	47.3	2.6	10.9	8.2
IV	23	40.8	1.8	6	10.5
V	11	39.9	3.6	26.5	5.0
VI	35	72.4	2.1	20.8	15.9
VII	6	34.7	5.7	24.7	2.7
VIII	8	11.7	1.4	3.5	3.6
IX	25	64.0	2.5	16.5	11.4
X	5	34.1	6.8	27.6	2.2
Total	219	509.7	2.3		

^a^Maximum gap within the LG. ^b^Percentage of SNP loci contained within the LG

### QTL analysis

Composite interval mapping with a sliding window size of 10 cM detected QTL for sweetness and the other fruit quality traits within the two harvest years (2016 and 2017). In total, 21 QTL were distributed across nine LG (all except LG VIII) (Fig. 1). The proportion of phenotypic variance explained by a single QTL ranged from 3.1 to 19.8% (Table 2). The highest percentage of explained phenotypic variance by a single QTL was observed for fruit length (19.8%), followed by fruit width (19.5%) and fruit firmness (15.5%, LG I; Year 2017), while the lowest was detected for fruit firmness (3.1%, LG IX; Year 2016). In general, QTL for individual traits were observed at a similar map location in both 2016 and 2017. The number of QTL detected for each trait varied from 2 to 5 loci. The largest number of QTL was observed for fruit firmness (5 loci). In contrast, the lowest number of QTL was observed for flesh thickness (2 loci), followed by flesh sweetness (3 loci). The relationship among fruit quality traits was evidenced by co-location of QTL on LG I, III, IV, VI, IX and X. For example, QTL for flesh sweetness were clustered together with QTL for fruit firmness and fruit length on LG III. Also, QTL for skin freckle were clustered with QTL for fruit firmness on LG IX. QTL for fruit size characteristics (fruit weight, length and width) and fruit firmness clustered on several LG including I, IV, VI, IX and X.

**Table 2:** Locations, effects and significance of QTL detected for papaya fruit quality traits

Trait	QTL	LG	QTL Peak^a^	LOD	Interval ^(cM)^	Closest marker	Pos^b^	%Var^c^	*P* ^d^	AE^e^
Flesh sweetness	SWE_2016	7	33.7	15.1	31.7–34.6	sCT_80_62821387	33.6	11.6	**	−0.64
	SWE_2017	3	6.2	7.0	0.0–11.2	sCT_6_9957924	6.2	3.3	–	-0.46
		7	4.0	13.2	0.0–14.0	sCT_12_17261968	0.0	10.6	***	−0.68
Fruit weight	WEI_2016	1	52.9	6.2	48.8–56.8	sCT_20_25190533	52.8	14.2	–	148.33
		4	18.6	4.2	14.7–21.0	sCT_6_10945637_2	19.1	12.7	**	71.29
		10	15.6	3.2	0.0–26.5	sCT_48_47598629	6.5	4.6	–	78.21
	WEI_2017	1	51.1	5.5	45.8–55.8	sCT_20_25182916	51.1	13.9	–	140.40
		4	17.6	5.6	16.0–20.6	sCT_6_10847720	17.6	11.3	**	129.78
		10	15.6	3.6	0.0–26.5	sCT_48_47598629	6.5	5.2	–	93.81
Fruit length	LEN_2016	1	30.4	5.8	19.3–39.2	sCT_42_43407698	36.2	17.2	–	1.93
		6	47.0	8.1	0.0–12.2	sCT_50_48742082	47.0	12.3	*	0.70
	LEN_2017	1	29.4	5.9	15.3–49.8	sCT_42_43407698	36.2	19.8	–	1.84
		3	4.3	4.5	43.1–50.0	sCT_6_9994764_1	3.3	5.8	*	0.32
Fruit width	WID_2016	4	13.4	17.3	12.8–16.0	sCT_6_10818885	13.9	19.5	**	0.39
	WID_2017	1	99.9	15.1	94.2–105.7	sCT_43_43923875	99.8	6.5	–	0.34
		4	13.9	20.7	13.8–17.1	sCT_6_10818885	13.9	12.5	**	0.45
		6	49.7	13.7	45.3–55.8	sCT_50_48651826	50.0	3.3	**	−0.17
		9	51.5	11.7	47.1–55.3	sCT_37642_123643261	50.4	6.8	***	−0.65
Skin freckle	FRE_2016	2	25.0	7.6	17.0–27.0	sCT_3_6222563	28.0	7.45	*	−0.23
		9	10.5	5.5	0.0–32.2	sCT_33_36735495	9.5	8.5	*	−0.41
	FRE_2017	2	26.0	5.6	15.0–27.0	sCT_3_6222563	28.0	6.27	*	−0.03
		6	39.1	5.1	28.5–49.7	sCT_16_21825379_2	39.1	3.23	**	0.47
		9	8.2	4.3	0.0–32.2	sCT_33_36735495	9.5	7.45	*	−0.25
Fruit firmness	FIR_2016	1	80.4	8.7	75.0–85.8	sCT_751_106036863	81.0	16.3	*	−0.74
		3	6.2	7.7	0.0–10.2	sCT_6_9957924	6.2	6.1	*	0.10
		5	13.3	3.4	0.0–22.8	sCT_7_12095557	6.3	4.3	–	0.38
		9	16.5	5.1	11.4–23.4	sCT_33_36735495	9.5	3.1	–	0.30
		10	4.9	5.2	0.0–17.5	sCT_48_47598629	6.5	4.4	*	−0.28
	FIR_2017	1	82.0	9.3	78.3–86.1	sCT_751_106036864	82.8	15.5	*	−0.69
		3	6.2	6.4	0.0–12.2	sCT_6_9957924	6.2	7.7	*	0.16
		9	16.5	5.0	11.4–23.4	sCT_33_36735495	9.5	6.8	–	0.27
		10	4.9	4.7	0.0–17.5	sCT_48_47598629	6.5	5.8	*	−0.25
Flesh thickness	THI_2016	4	3.0	11.7	0.0–5.5	sCT_6_10509125	3.1	8.4	**	0.26
		9	34.9	9.9	32.5 39.7	sCT_114_74895785	34.9	4.3	–	0.61
	THI_2017	9	44.1	13.4	34.8–50.4	sCT_34334_12288738	44.5	6.5	–	0.60

^a^Position of QTL peak (cM). ^b^Closest marker position. ^c^Percentage of explained phenotypic variance. ^d^Significance in single marker analysis at *p*-value ≤0.05 (*), 0.01 (**) and 0.001 (***) determined by Kruskal-Wallis test. ^e^Additive effect (where a positive value indicates that alleles from RB2 contributed to increasing the trait score and a negative value indicates that alleles from Sunrise Solo contributed to increasing the trait score)

### Candidate genes for flesh sweetness and other fruit quality traits

The regions within major QTL intervals were annotated according to the ‘Sunup’ reference genome. Three candidate genes responsible for regulation of developmental growth (non-canonical poly(A) RNA polymerase and KIN17-like protein (accession number: XP_021903675 and XP_021907879) and protein transmembrane transporter activity (accession number: XP_021887112) were detected within the flesh sweetness QTL peaks (Additional file 7: Table S5). The regions of fruit weight, length and width QTL contained candidate genes involved in cell wall organisation (protein trichome birefringence-like 12 and fatty acid amide hydrolase-like), protein metabolic process (glutamate receptor 3, IST1-like protein, prolyl 4-hydroxylase 9 and bifunctional nuclease 2) and carbohydrate metabolic process (exopolygalacturonase and NAC domain-containing protein 41). The previously identified *Carica papaya* chromosome Y sequence on LG1 [15, 41] was also found near fruit length QTL. Two candidate genes (Ultraviolet-B receptor and putative disease resistance protein RGA1) were observed within skin freckle QTL. Fruit firmness QTL regions contained one candidate gene encoding pectin catabolic process (pectin acetyl esterase 12-like) and three candidate genes related to transcription factor activity (UPF0553 protein-like, DNA-directed RNA polymerase III subunit 1 and MYB-like protein X). Candidate genes responsible for lignin biosynthetic processes and ethylene-activated signaling pathways were identified within the QTL regions for flesh thickness.

## Discussion

### SNP discovery

For the first time, genotyping-by-sequencing (GBS) was successfully used to develop a SNP linkage map and identify key genomic locations underlying flesh sweetness and other fruit quality traits in papaya. Also, in conjunction with the existing reference genome, several QTL-linked SNP loci were associated with putative candidate genes.

The frequency and number of SNPs obtained by GBS in the ‘RB2’ x ‘Sunrise Solo’ population was comparable to that reported in sweet cherry [25], zucchini [48] and tomato [11] using the same approach. However, the majority of identified SNPs (96%) were excluded from the map construction, resulting in a far lower number of SNPs in the final linkage map than in the previously mentioned ones. After stringent filtering all loci with minimum read depth, missing data and identifiable parental alleles, the number of SNP loci reduced below that which has been typically reported in other species. In zucchini, the work of Montero-Pau et al. [48] revealed 25% (16,222 markers) of validated SNPs derived from GBS. Approximately 13% of high quality SNPs (3125 markers) were discovered in tomato by GBS approach [11]. The variation in percentage of validated SNPs obtained in the current study and other studies could be attributed to a number of factors including selection of restriction enzymes and sequencing depth, sample library preparation, genetic background of plant materials and condition of data analysis [16, 43, 71]. Strategies such as adjusting the level of multiplexing, changing the choice of restriction enzyme(s) and increasing sequencing depth could be investigated to increase the capture rate of SNPs in the population [4, 71]. Among these factors, the condition of GBS data analysis was reported as a major impact on the amount and quality of the resulting genotypic information [71]. The number of called SNPs, missing data and genotypic accuracy varied vastly due to the choice of an analytical method and the reference genome used for SNP-mapping [4, 71]. Under the condition used in this study, the detection of a polymorphism was reliant on the existing ‘Sunup’ reference genome [46], which was incomplete in terms of assembly contiguity, number of gap sequences and genome coverage (~ 75%). It is entirely possible that the quality of the reference genome affected the process of SNP-calling through inability to align raw sequencing output with the existing reference assembly and resulted in the relatively low number of validated SNP for mapping. In future, high coverage genome sequences of both parents (‘RB2’ and ‘Sunrise Solo’; Genbank SRA accession: PRJNA507836) should be used as reference genomes for SNP-discovery and the mapping of their recombinants [29, 30, 39]. Alternatively, if a high quality reference genome is not available, a de novo SNP discovery approach could be considered (Described in Catchen et al., [9, 54, 60];).

### Linkage map construction

An extremely high percentage of marker segregation distortion was detected (76.4%, *P* < 0.05), consistent with previous studies such as Blas et al. [5] who reported 79% marker segregation distortion in a ‘Khaek Dum’ x ‘2H94’ cross population. Similarly, 66% segregation distortion was observed among markers in a ‘AU9’ and ‘Sunup’ cross population [15]. The underlying reasons for segregation distortion include genetic interaction among loci [42], the predominance of parental or recombinant genotypes in the population, environmental factors and experimental errors [2, 75, 76]. The high number of distorted loci in this study is likely attributed to dominance of one parental genotype, with twice as many maternal (‘RB2’) than paternal (‘Sunrise Solo’) alleles identified, as well as missing genotypic data [31].

Although the final map was not as dense as the linkage map of Blas et al. [5], the marker placement and alignment was robust with adequate resolution for QTL mapping [19]. The quality and applicability of a linkage map with similar density was demonstrated previously by Bielenberg et al. [4] who used 33 SSR and 201 SNP markers identified from GBS pipeline to construct a genetic map with an average marker interval of 2.85 cM to detect QTL for chilling requirement and bloom date in peach.

The chromosome-specific cytogenetic markers were developed and merged with linkage groups of papaya using the integrated technique of fluorescence in situ hybridisation (FISH) and BAC clones harboring mapped SSR markers as probes [74]. Nine linkage groups was proposed and corresponded to the haploid number of papaya chromosomes. However, we are unable to integrate these maps as there are no anchor markers shared among them. The reason being that different parents were used to construct the mapping populations.

### QTL and candidate genes for individual fruit quality traits

QTL mapping is useful for dissecting the genetic components of complex traits [3]. The QTL analysis in the F2 population of ‘RB2’ x ‘Sunrise Solo’ detected 21 QTL affecting fruit quality in papaya. Most of the traits were associated with two to five QTL, indicating their polygenic nature [26, 45, 77]. Ten of the 21 QTL detected in this study had > 10% effect on the phenotypic variance and were characterised as a major QTL [69]. Several of these were stable over two harvest years, indicating their potential for investigation in future trait selection.

Co-location of QTL for different fruit quality traits was indicated in several genome regions as similarly reported in other species [13, 80]. QTL identified in the same location may contain shared and/or distinct genes with potential pleiotropic effects. Multiple QTL with large effects were shown responsible for fruit sweetness in other species including in peach [22] and apple [26]. These were located close to QTL associated with fruit weight and size but with opposite allelic effects, again suggesting pleiotropic activity [22, 26, 32]. Further studies with near-isogenic lines are required to tease apart the QTL in the current study and to identify possible individual candidate genes for further functional validation of association with each of the specific traits.

In the present study, the exploration of genetic variation and transferability of key fruit quality traits within the parental and progeny population of ‘RB2’ x ‘Sunrise Solo’ genotypes indicated high heritability (> 60%) for flesh sweetness, fruit width and fruit firmness (Additional file 2: Table S2). This confirmed the high heritability of several fruit traits previously described for flesh sweetness, flesh colour, flesh firmness, fruit firmness and fruit size in papaya [53, 63] and other fruit crops [7, 58]. Whereas, the rest of traits showed low to moderate heritability (30–60%) and the lowest heritability was found in fruit weight (32%). The likelihood of success in QTL identification and mapping depends on the heritability of the trait, its genetic nature (dominant, recessive or additive) and the number of genes involve [1]. Theoretically, identification of QTL for high heritability traits should be easier to detect and likely to explain more of the phenotypic variation as they should be less influenced by environmental factors [27]. This assumption appeared to be true in the case of flesh sweetness, fruit width and fruit firmness. The QTL analysis clearly identify their major governing genetic loci across two harvest seasons and with relatively large likelihood (11.6 to 19.5%). Meanwhile, the identification of QTLs of traits with low to moderate heritability also revealed QTLs with large effect in fruit weight and fruit length. It is possible that these traits are closely correlated to traits with high heritability, which are fruit width and fruit firmness, therefore, the clustering of QTLs among these fruit morphology traits may result in large effect size estimates due to the co-location of the detected QTLs. In contrast, most of the QTL identified for skin freckle and flesh thickness were minor QTL. These occurrences are commonly observed for QTL of fruit quality in other species, reflecting their polygenic nature and the high influence of environmental conditions [5, 12, 26, 32].

Flesh sweetness is quantitatively inherited with many studies revealing multiple QTLs responsible including in Rosaceae such as peach, apple and strawberry [22, 26, 38]. The QTLs for flesh sweetness were detected across multiple genome locations with a range of effect (up to 84%). Several QTLs were associated with the sucrose synthase gene (*SUSY1*) family and a gene encoding vacuolar H + -pyrophosphatase which catalyses solute accumulation [22, 28]. The current study is the first for papaya and proposes that flesh sweetness is under polygenic control in the cross between ‘RB2’ x ‘Sunrise Solo’. At least two genomic regions were identified and associated with genes responsible for growth development and protein transmembrane transporter activity. As expected, alleles of ‘Sunrise Solo’ (the sweeter parent) contributed to an increase of sweetness in the progeny. The sweetness trait-associated major QTL on group VII that contained growth development and protein transmembrane transporter activity genes directly linked with SNP loci; sCT_80_454708 and sCT_12_1083429 require further exploration. These should be assessed for stability and functional association potentially through targeted amplification across a wider range of genotypes and reverse genetics approaches [50, 70].

The genetic governance of fruit weight, length and width has been widely studied in many fruit crops including tomato [40], pepper [81] and melon [28]. Accordingly, members of the *ovate, sun and fw2.2* gene families were detected within the related QTL [40, 81]. In papaya, QTL for fruit weight and size were previously identified in F_2_ populations of ‘Sunrise Solo’ x Line 356 [64] and ‘Khaek Dum’ x ‘2H94’ [5] but as in the current study, were not associated with any *ovate, sun or fw2.2* genes [5]. Rather, fruit weight, length and width QTL on LGI in this study were in close proximity to a papaya male-specific region previously associated with elongated fruit. The four SNP markers, sCT_6_2754743, sCT_6_2392635, sCT_50_1447788 and sCT_6_2331252, that were mapped within 1 cM of the major QTL for these traits should be explored further for functional association.

Skin freckle is one of the major issues affecting fruit quality of papaya and its genetic basis is not been well understood. Eloisa et al. [20] reported that skin freckle of papaya fruit was highly influenced by weather condition, fruit growth and fruit sugar content. In this present study, QTL analysis for skin freckle did not detect any relationship between skin freckle and flesh sweetness QTLs, however co-localisation of QTLs for skin freckle, fruit firmness, fruit width and length was observed. Indeed, skin freckle was shown to be conditioned by several minor QTLs on LG II, VI and IX (each accounted for 3.23 to 8.5%). However, these accounted for relatively little of the trait variation again likely due to the missing genome coverage and potential epistatic interactions that reduces detection of small effect QTLs [62]. Therefore, targeting the three loci identified in this study may be insufficient for improving skin quality of papaya.

The genetic basis of variation in fruit firmness and flesh thickness has been studied most extensively in tomato, cucurbits and apple [13, 36, 68, 78]. Most QTLs for fruit firmness and flesh thickness have been described with association with ethylene response factor and members of expansine, pectin methylesterase and protein-lysine methyltransferase gene families [14, 78]. Similarly, genes encoding pectin catabolic process and ethylene-activated signalling pathway were found in this study within locations of stable QTLs in ‘RB2’ x ‘Sunrise Solo’ mapping, suggesting similar functions for these genes in papaya. Five markers (sCT_751_466, sCT_751_404, sCT_6_237757, sCT_48_1243956, sCT_6_1666511) associated with the QTLs for fruit firmness and flesh thickness were mapped within a 3 cM window. These markers may be useful for future breeding selection.

## Conclusions

In summary, this study demonstrated the use of GBS technology for efficient QTL detection in papaya (F_2_ population of ‘RB2’ x ‘Sunrise Solo’). The SNP based genetic map and QTL for flesh sweetness, fruit weight, width, length, skin freckle, firmness and flesh thickness detected in two successive years and associated SNPs provide target regions for candidate gene exploration and selective marker development.

## Methods

### Plant materials and phenotyping of fruit quality characters

Parental lines and 226 segregating F_2_ progeny of the ‘RB2’ x ‘Sunrise Solo’ cross were planted in Mareeba, Australia and evaluated for fruit quality traits across two harvests; in December 2016 and April 2017. The two parental lines used in the experiments are Australian commercial varieties. These were produced by Papaya Seed Australia who provided permission for their use in this scientific research. Plant experiment was performed in the School of Environment and Science, Griffith University, according to a plant protocol approved by the Research Committee of Griffith University. At each harvest, three fruit from each individual plant were harvested and measured for quantitative phenotypic data of flesh sweetness, fruit weight, fruit length, fruit width skin freckle, flesh thickness and fruit firmness in accordance with the methods outlined in the Papaya Handbook ([49], Additional file 1: Table S1, Additional file 2: Table S2, Additional file 3: Figure S1).

### Genotyping-by-sequencing (GBS) and SNP identification

A GBS approach was used to detect single nucleotide polymorphisms (SNP) between the parental and among the F_2_ genomes. For this, gDNA was extracted using the modified CTAB protocol of Dellaporta et al. [17] from individual leaf samples of one-year-old trees of parents and F_2_ progeny. Quality and quantity of gDNA was assessed with a NanoDrop 1000c (Thermo Fisher Scientific, Australia) and diluted to 100 ng/μl. DNA samples were sent for GBS at the Australian Genome Research Facility, Melbourne, Australia, using a ddRAD-based library preparation protocol, as described in Peterson et al. [56]. The DNA was digested using a combination of restriction enzymes (*PstI* and *MseI*) and only tags with both RE sites (one at each end) were selected for library preparation and sequenced on an Illumina HiSeq2500 sequencing platform, producing 100 bp single-end reads. Parental DNA was sequenced thrice and F_2_ individuals were sequenced once each to generate SNP catalogues (Genbank SRA accession: PRJNA544124). Raw GBS reads were de-multiplexed and sorted according to their barcoded sequences using Stacks software v1.46 [10]. The resultant filtered reads (high-quality sequences from each sample) were aligned to the papaya reference genome ‘Sunup’ variant [46] using Bowtie2 version 2.3.2 [37].

SNP identification was carried out using gstacks command in Stacks2 v2.00beta5 [10] to obtain only bi-allelic SNPs polymorphic between the parents. Subsequently, SNPs were filtered using SnpSift v4.3p [61] with the following parameter settings: Minimum read depth larger than five (DP > 5) and Phred genotype quality score of more than 20 (GQ > 20). In addition, the genomic positions of the SNPs were determined according to the ‘Sunup’ reference genome [46] and used to assign the SNP ID. Further SNP filtration was performed using in-house R script [59]. Loci with > 80% missing data were discarded. The imputation of missing genotypes was performed using LinkImputeR v1.1.1 [47] and resulted in 1701 high quality SNP loci for linkage map construction.

### Linkage map construction

An initial linkage map was constructed after removal of duplicated and monomorphic markers using Onemap R package [44] and with a logarithm of the odds (LOD) threshold of 5.0 and a maximum recombination fraction (max.rf) threshold of 0.25. Subsequently, linkage groups (LG containing less than four loci and any unlinked markers were excluded. The Rapid Chain Delineation (RCD) algorithm was used to order markers within each LG [18]. Then, 10 equally spaced markers in a LG were selected to create a framework of ordered markers using the “make_seq” and “compare” functions. The remaining markers were added to the framework with the “order_seq” function with the lowest threshold for a positioning marker of LOD 3.0. The combination of markers was then inspected (within a window size of four markers) using the “ripple” function to obtain the final marker order. Map distance in centiMorgans (cM) was estimated by the Kosambi mapping function [35].

The final linkage map was created after removal of markers with significant deviation from the expected segregation ratio using the “select_segreg” function and the remaining markers were again clustered into LG and ordered as described above. Initial and final maps were visualised using Mapchart [73]. The R/qtl package [8] was used to generate input files for QTL analyses.

### QTL mapping

QTL analyses were performed using WinQTLCart software version 2.5 [75, 76].. First, single marker analysis was performed using the nonparametric Kruskal-Wallis test to individually associate markers and traits. Then, interval mapping analyses were undertaken to locate QTL position on the map. Composite Interval Mapping (CIM) was selected as the mapping method for sensitivity and to enable multiple potential QTL detection for each trait. The standard CIM Model was used (model number 6 with a value of 5 for control markers and a forward regression). The LOD threshold was determined by a 1000 permutation test with a significance level (*p*) set at 0.05. Two sets of fruit quality trait data (harvest years 2016 and 2017) were analysed separately for all tested traits to assess QTL stability and detect additional seasonal QTL. QTL that had a LOD > 3 and a phenotypic variance contribution > 10% were classified as major QTL [69]. In addition, a QTL that appeared in both harvests was classified ‘stable’. Additive effects were estimated where a positive value indicated that alleles contributed from ‘RB2’ increased the trait score and a negative value indicated that alleles contributed from ‘Sunrise Solo’ increased the trait score.

### Identification of linked markers and putative candidate genes

Significant association of SNP marker with QTL peak region was determined by the Kruskal-Wallis test with 95% confidence (*p* ≤ 0.05). Subsequently, the gene annotation database from the ‘Sunup’ reference genome (http://www.plantgdb.org/XGDB/phplib/download.php? GDB=Cp) together with the database of the National Centre for Biotechnology Information (NCBI; https://blast.ncbi.nlm.nih.gov/Blast.cgi) and Phytozome (https://phytozome.jgi.doe.gov/pz/portal.html) were utilised to search for location information of the identified markers and candidate genes within the major QTL peak regions. Flanking sequences at both sides of the significant SNP positions were used as queries in BLAST searches against the DNA database and the *Carica papaya* genome sequence, ASGPBv0.4 with an E-value ≤1e^− 15^, identity ≥70% and coverage ≥50%. Gene Ontology (GO) terms associated with each BLAST hit were annotated using the GO Consortium BLAST server (http://www.geneontology.org).

## Supplementary information

**Additional file 1:** **Table S1.** Mean and standard deviation of fruit quality traits of parental lines and their F1 and F2progeny population in 2016 and 2017. This table presents phenotypic evaluation of seven fruit quality traits.

**Additional file 2:** **Table S2.** Phenotypic variances by generation in 2016 and 2017 and heritability estimates of each fruit quality trait.

**Additional file 3:** **Figure S1.** Phenotypic variation of fruit quality traits (A-G) among parents, F1 and F2 populations. Mean and median values are represented by black solid lines (−) and red cross (+), respectively in the interior of each box area. The mid-parent values are indicated by horizontal dashed lines.

**Additional file 4:** **Table S3.** Summary of initial linkage map from F2 population of ‘RB2’ x ‘Sunrise Solo’.

**Additional file 5:** **Table S4.** Summary of SNPs markers and segregation.

**Additional file 6:** **Figure S2.** Genetic map of ‘RB2’ x ‘Sunrise Solo’ and QTL for fruit quality traits. The LGs resulted from initial map and final map were labelled by LG1-LG23 and I-X, respectively. The left pane indicates the genetic map position in cM of each SNPs. Homology between both maps was highlighted in turquoise. Colour bars on the right of final map indicate QTL position and LOD interval at 95% confidence; where flesh sweetness (SWE) – red; fruit weight (WEI)-brown; fruit length (LEN)-green; fruit width (WID)-olive; skin freckle (FRE)-pink; flesh thickness (THI)-black; fruit firmness (FIR)-blue. Data from harvest year 2016 and 2017 are represented in solid and diagonal-stripe bar, respectively.

**Additional file 7:** **Table S5.** Associated SNPs and candidate genes for flesh sweetness and other fruit quality traits.

## Data Availability

The sequence data generated during this study have been deposited in Genbank repository with accession code PRJNA507836 https://www.ncbi.nlm.nih.gov/sra/PRJNA507836 and PRJNA544124 https://www.ncbi.nlm.nih.gov/sra/PRJNA544124. The other data that support the findings of this study are available within the article and its supplementary information files.

## Abbreviations

AFLP: Amplified fragment length polymorphism
CIM: Composite Interval Mapping
cM: centiMorgan
DP: Minimum read depth
GBS: A genotyping-by-sequencing
GO: Gene Ontology
GQ: Phred genotype quality score
LG: Linkage groups
LOD: Logarithm of the odds
MAS: Marker-assisted selection
QTL: Quantitative trait loci
RAPD: Random amplified polymorphic DNA
RCD: Rapid Chain Delineation
SNP: Single nucleotide polymorphism
SSR: Simple sequence repeat
