# Supplementary material for: Linkage mapping and quantitative trait loci analysis of sweetness and other fruit quality traits in papaya
Source: BMC Plant Biol. 2019 Oct 26;19:449. doi: 10.1186/s12870-019-2043-0 (PMC6815024; doi:10.1186/s12870-019-2043-0)
Supplement: Supplementary file 1 — Additional file 1: Table S1. Mean and standard deviation of fruit quality traits of parental lines and their F1 and F2progeny population in 2016 and 2017. This table presents phenotypic evaluation of seven fruit quality traits. [file 12870_2019_2043_MOESM1_ESM.pdf]

Table S1: Mean and standard deviation of fruit quality traits of parental lines and their F_1 and F_2 progeny population in 2016 and 2017

Traits	Year	'RB2'	'Sunrise Solo'	F_1	F_2
**Flesh sweetness**	2016	8.54 ± 0.52	12.90 ± 0.39	10.53 ± 0.89	10.26 ± 1.23
	2017	9.12 ± 0.53	13.63 ± 0.58	11.23 ± 0.97	10.69 ± 1.55
	Combined [/1]	8.83 ± 0.53 [D]	13.26 ± 0.49 [A]	10.88 ± 0.93 [B]	10.48 ± 1.39 [C]
	P-value [/2]	**0.001**	**< 0.0001**	**< 0.0001**	**< 0.0001**
**Fruit weight**	2016	1273.90 ± 314.04	483.68 ± 145.78	810.28 ± 205.05	859.02 ± 277.17
	2017	1341.92 ± 338.33	542.24 ± 121.73	844.03 ± 202.92	888.76 ± 274.19
	Combined [/1]	1307.91 ± 326.18 [A]	512.96 ± 133.75 [C]	827.16 ± 203.98 [B]	873.89 ± 275.68 [B]
	P-value [/2]	0.364	0.056	0.231	0.033
**Fruit length**	2016	19.67 ± 1.80	13.91 ± 1.52	16.57 ± 1.93	16.63 ± 2.69
	2017	20.37 ± 1.54	14.66 ± 1.07	16.84 ± 1.71	16.66 ± 2.45
	Combined [/1]	20.02 ± 1.67 [A]	14.29 ± 1.29 [C]	16.71 ± 1.82 [B]	16.64 ± 2.57 [B]
	P-value [/2]	0.108	**0.009**	0.554	0.894
**Fruit width**	2016	10.67 ± 0.95	7.46 ± 0.91	9.41 ± 0.83	9.70 ± 1.50
	2017	11.79 ± 1.10	8.49 ± 0.48	9.86 ± 0.83	10.59 ± 1.70
	Combined [/1]	11.23 ± 1.02 [A]	7.98 ± 0.70 [D]	9.63 ± 0.86 [C]	10.14 ± 1.60 [B]
	P-value [/2]	**< 0.0001**	**< 0.0001**	**< 0.0001**	**< 0.0001**
**Skin freckle**	2016	1.00 ± 0.00	4.88 ± 0.33	2.08 ± 1.20	2.15 ± 1.37
	2017	1.00 ± 0.00	4.69 ± 0.47	1.79 ± 1.01	1.69 ± 1.13
	Combined [/1]	1.00 ± 0.00 [C]	4.78 ± 0.40 [A]	1.94 ± 1.10 [B]	1.92 ± 1.25 [B]
	P-value [/2]	> 0.99	**0.027**	0.106	**< 0.0001**
**Fruit firmness**	2016	3.51 ± 0.34	1.45 ± 0.25	2.18 ± 0.78	2.54 ± 1.10
	2017	3.67 ± 0.36	1.50 ± 0.32	2.27 ± 0.75	2.60 ± 1.03
	Combined [/1]	3.59 ± 0.35 [A]	1.48 ± 0.29 [D]	2.22 ± 0.76 [C]	2.57 ± 1.07 [B]
	P-value [/2]	0.053	0.657	0.231	0.113
**Flesh thickness**	2016	3.49 ± 0.55	1.61 ± 0.21	2.19 ± 0.41	2.43 ± 0.58
	2017	3.51 ± 0.47	1.75 ± 0.21	2.25 ± 0.36	2.49 ± 0.52
	Combined [/1]	3.50 ± 0.51 [A]	1.68 ± 0.21 [D]	2.22 ± 0.76 [C]	2.46 ± 0.55 [B]
	P-value [/2]	0.842	**0.002**	0.373	**0.007**

[/1] Different letters within a row indicate significant differences for trait values among populations determined with Tukey's HSD test

[/2] Bolded figures represent significant differences in the mean values observed among 2016 and 2017 harvests at $p < 0.05$ determined with the Mann-Whitney test.
