# Supplementary material for: Linkage mapping and quantitative trait loci analysis of sweetness and other fruit quality traits in papaya
Source: BMC Plant Biol. 2019 Oct 26;19:449. doi: 10.1186/s12870-019-2043-0 (PMC6815024; doi:10.1186/s12870-019-2043-0)
Supplement: Supplementary file 2 — Additional file 2: Table S2. Phenotypic variances by generation in 2016 and 2017 and heritability estimates of each fruit quality trait. [file 12870_2019_2043_MOESM2_ESM.pdf]

Table S2: Phenotypic variances by generation in 2016 and 2017 and heritability estimates of each fruit quality trait

Traits	Year	σ^2 (RB2) /1	σ^2 (Solo) /1, /7	σ^2 (F_1) /1	σ^2 (F_2) /1	σ^2 (Phe) /2	σ^2 (E) /3	σ^2 (G) /4	H^2 /5	H^2 % /6
**Flesh sweetness**	2016	0.26	0.15	0.79	1.52	1.52	0.5	1.02	0.67	67.30%
	2017	0.27	0.33	0.93	2.39	2.39	0.62	1.77	0.74	74.20%
	Combined	0.26	0.33	0.95	2	2	0.62	1.38	0.69	68.90%
**Fruit weight**	2016	94967.2	20809.39	41459.63	76707.52	76707.52	49673.96	27033.56	0.35	35.20%
	2017	110227.78	14509.09	40605.29	75069.65	75069.65	51486.86	23582.79	0.31	31.40%
	Combined	103754.1	18516.68	41317.23	76109.77	76109.77	51226.31	24883.46	0.33	32.70%
**Fruit length**	2016	3.13	2.26	3.69	7.22	7.22	3.19	4.03	0.56	55.80%
	2017	2.3	1.11	2.87	5.98	5.98	2.29	3.7	0.62	61.80%
	Combined	2.84	1.83	3.3	6.6	6.6	2.82	3.79	0.57	57.40%
**Fruit width**	2016	0.86	0.8	0.69	2.24	2.24	0.76	1.48	0.66	66.10%
	2017	1.17	1	0.76	2.4	2.4	0.92	1.48	0.62	61.50%
	Combined	1.33	0.78	0.77	2.76	2.76	0.91	1.84	0.67	66.80%
**Skin freckle**	2016	0	0.11	1.41	1.86	1.86	0.73	1.13	0.61	60.70%
	2017	0	0.21	1	1.28	1.28	0.55	0.73	0.57	57.00%
	Combined	0	0.17	1.23	1.63	1.63	0.66	0.97	0.6	59.70%
**Fruit firmness**	2016	0.11	0.06	0.6	1.21	1.21	0.34	0.87	0.72	71.70%
	2017	0.13	0.1	0.55	1.06	1.06	0.33	0.73	0.69	68.60%
	Combined	0.13	0.08	0.58	1.14	1.14	0.34	0.8	0.7	70.10%
**Flesh thickness**	2016	0.3	0.04	0.17	0.34	0.34	0.17	0.17	0.5	49.70%
	2017	0.21	0.04	0.12	0.27	0.27	0.13	0.14	0.53	53.10%
	Combined	0.25	0.05	0.15	0.3	0.3	0.15	0.15	0.51	50.80%

/1 Generation variances (σ^2) in 'RB2', 'Sunrise Solo', F_1 and F_2 population.

/2,/3,/4 Phenotypic [σ^2(Phe)], environmental [σ^2(E)] and genetic [σ^2(G)] components.

/5,/6 Broad sense heritability (H^2) and percentage of heritability and (H^2%) of each trait.

Estimation of variances components, board sense heritability and percentage of heritability were calculated using formulas derived by Warner (1952). Heritability was classified as low (below 30%), moderate (30-60%) or high (above 60%) (Kumar et al. 2016)

/7 *C. papaya* 'Sunrise Solo'
