# Supplementary material for: Linkage mapping and quantitative trait loci analysis of sweetness and other fruit quality traits in papaya
Source: BMC Plant Biol. 2019 Oct 26;19:449. doi: 10.1186/s12870-019-2043-0 (PMC6815024; doi:10.1186/s12870-019-2043-0)
Supplement: Supplementary file 3 — Additional file 3: Figure S1. Phenotypic variation of fruit quality traits (A-G) among parents, F1 and F2 populations. Mean and median values are represented by black solid lines (−) and red cross (+), respectively in the interior of each box area. The mid-parent values are indicated by horizontal dashed lines. [file 12870_2019_2043_MOESM3_ESM.pdf]

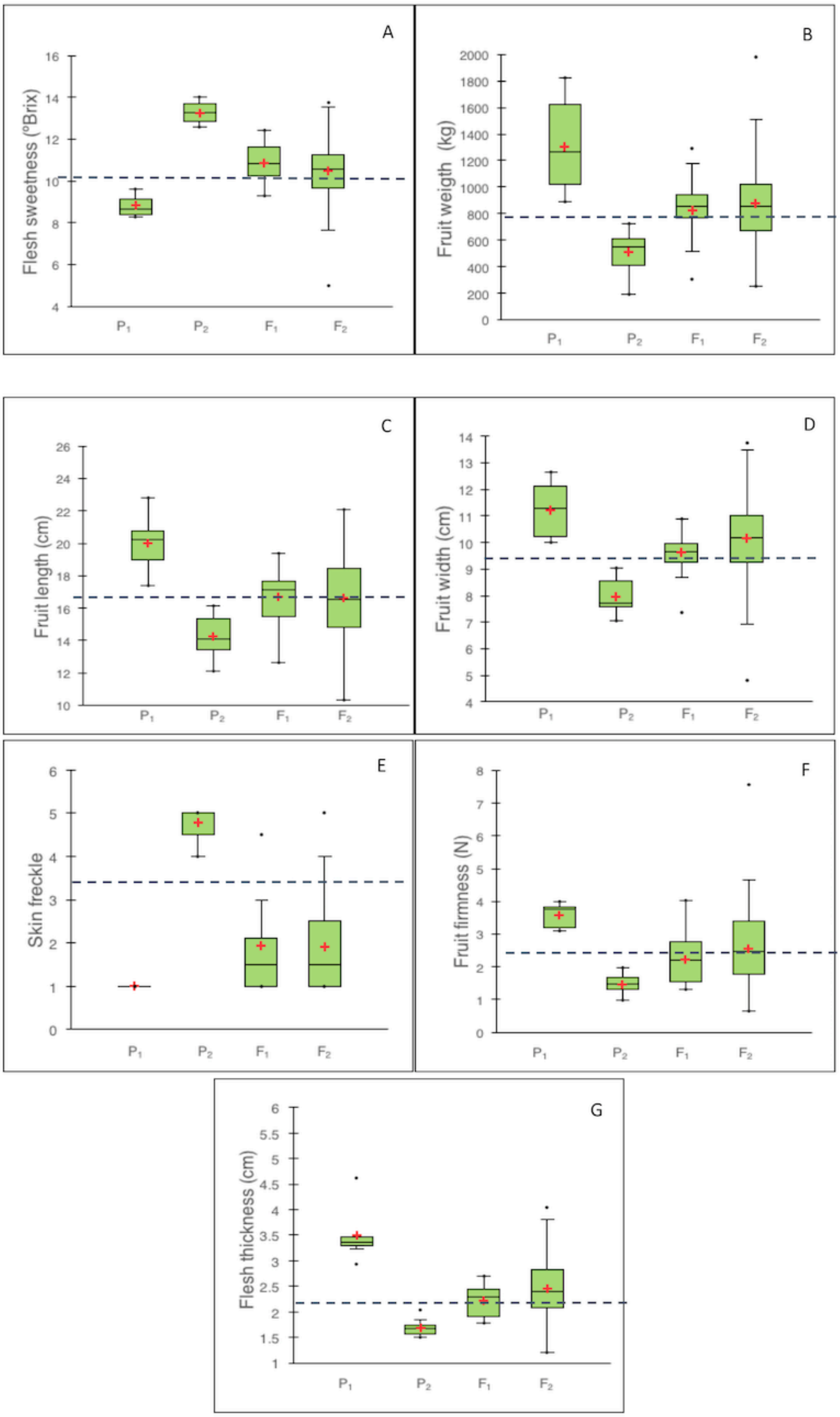

Figure S1: Phenotypic variation of fruit quality traits (A-G) among parents, F1 and F2 populations. Mean and median values are represented by black solid lines (-) and red cross (+), respectively in the interior of each box area. The mid-parent values are indicated by horizontal dashed lines.
