# Supplementary material for: Linkage mapping and quantitative trait loci analysis of sweetness and other fruit quality traits in papaya
Source: BMC Plant Biol. 2019 Oct 26;19:449. doi: 10.1186/s12870-019-2043-0 (PMC6815024; doi:10.1186/s12870-019-2043-0)
Supplement: Supplementary file 4 — Additional file 4: Table S3. Summary of initial linkage map from F2 population of ‘RB2’ x ‘Sunrise Solo’. [file 12870_2019_2043_MOESM4_ESM.pdf]

Table S3: Summary of initial linkage map from F_2 population of 'RB2' x 'Sunrise Solo'.

LG	No. of markers	Total length (cM)	Average marker interval (cM)	Gap[1/] (cM)	SDM[2/]	%SD[3/]
**1**	162	356.53	2.2	101.2	87	7.55
**2**	91	153.20	1.68	10.4	91	7.89
**3**	31	129.84	4.18	50.3	21	1.82
**4**	379	1093.11	2.88	26.6	379	32.87
**5**	30	138.16	4.6	68.6	12	1.04
**6**	48	211.02	4.39	104.5	25	2.17
**7**	21	52.41	2.49	30.0	10	0.87
**8**	115	270.53	2.35	59.5	90	7.81
**9**	48	70.39	1.46	7.8	48	4.16
**10**	27	100.71	3.73	62.8	21	1.82
**11**	20	47.43	2.37	7.9	12	1.04
**12**	26	98.50	3.78	41.3	26	2.25
**13**	42	64.15	1.52	8.5	42	3.64
**14**	10	74.21	7.42	26.9	10	0.87
**15**	27	48.53	1.79	6.6	2	0.17
**16**	22	41.65	1.89	11.9	22	1.91
**17**	7	46.52	6.64	27.7	2	0.17
**18**	10	20.71	2.07	7.7	10	0.87
**19**	12	19.64	1.63	8	12	1.04
**20**	6	21.6	3.6	6.7	6	0.52
**21**	10	10.15	1.01	2.2	10	0.87
**22**	4	13.31	3.32	7.2	4	0.35
**23**	5	14.63	2.92	10.6	5	0.43
**Total**	**1153**	**3096.93**	**2.7**	**-**	**882**	**76.4**

[1/] Maximum of average gap within LG.

[2/] Number of segregation distorted markers.

[3/] Percentage of segregation distorted marker out of total mapped markers.
