# Supplementary material for: Linkage mapping and quantitative trait loci analysis of sweetness and other fruit quality traits in papaya
Source: BMC Plant Biol. 2019 Oct 26;19:449. doi: 10.1186/s12870-019-2043-0 (PMC6815024; doi:10.1186/s12870-019-2043-0)
Supplement: Supplementary file 5 — Additional file 5: Table S4. Summary of SNPs markers and segregation. [file 12870_2019_2043_MOESM5_ESM.pdf]

Table S4: Summary of SNPs markers and segregation

Segregation	No. of makers	type	No. of markers	% marker
**Normal**	271	Mapped marker	219	17.1
		Unlinked marker	52	3.9
**Distorted**	882	Skew toward 'RB2'[1/]	187	21.3
		Skew toward 'Sunrise Solo'[1/]	98	11.2
		Other[2/]	597	46.5
**Total**	**1153**			

[1/] Segregation distorted markers which showed more than 75% of samples genotyped bias to one of the parents.

[2/] Segregation distorted markers which were not fit 1:2:1 expected ratio.
