# Supplementary material for: Linkage mapping and quantitative trait loci analysis of sweetness and other fruit quality traits in papaya
Source: BMC Plant Biol. 2019 Oct 26;19:449. doi: 10.1186/s12870-019-2043-0 (PMC6815024; doi:10.1186/s12870-019-2043-0)
Supplement: Supplementary file 6 — Additional file 6: Figure S2. Genetic map of ‘RB2’ x ‘Sunrise Solo’ and QTL for fruit quality traits. The LGs resulted from initial map and final map were labelled by LG1-LG23 and I-X, respectively. The left pane indicates the genetic map position in cM of each SNPs. Homology between both maps was highlighted in turquoise. Colour bars on the right of final map indicate QTL position and LOD interval at 95% confidence; where flesh sweetness (SWE) – red; fruit weight (WEI)-brown; fruit length (LEN)-green; fruit width (WID)-olive; skin freckle (FRE)-pink; flesh thickness (THI)-black; fruit firmness (FIR)-blue. Data from harvest year 2016 and 2017 are represented in solid and diagonal-stripe bar, respectively. [file 12870_2019_2043_MOESM6_ESM.pdf]

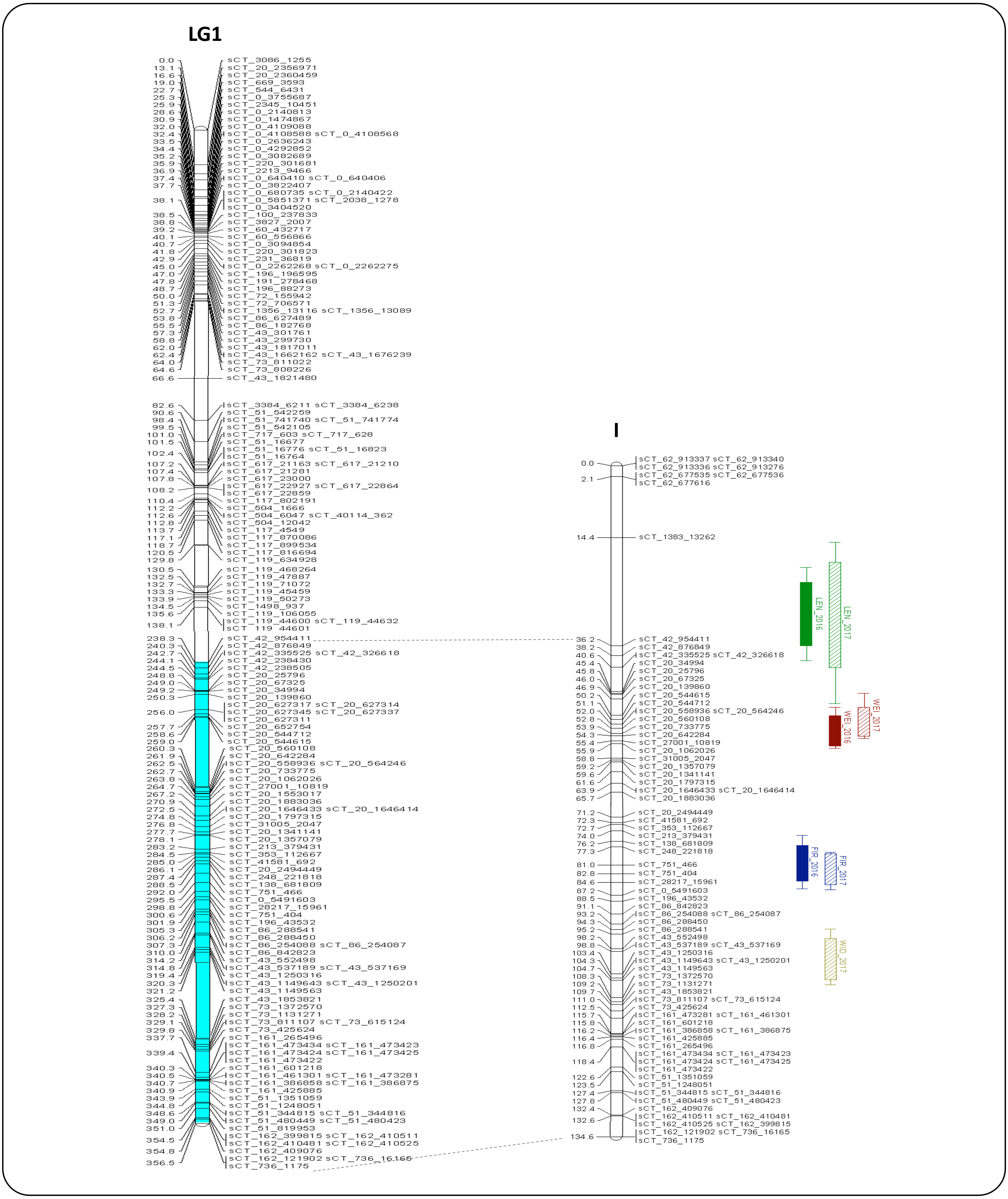

Figure S2 : Genetic map of 'RB2' x 'Sunrise Solo' and QTL for fruit quality traits.
The LGs resulted from initial map and final map were labelled by LG1-LG23 and I-X, respectively. The left pane indicates the genetic map position in cM of each SNPs. Homology between both maps was highlighted in turquoise . Colour bars on the right of final map indicate QTL position and LOD interval at 95% confidence; where flesh sweetness (SWE) – red ; fruit weight (WEI)-brown ; fruit length (LEN)-green ; fruit width (WID)-olive ; skin freckle (FRE)-pink ; flesh thickness (THI)-black ; fruit firmness (FIR)- blue . Data from harvest year 2016 and 2017 are represented in solid and diagonal-stripe bar, respectively.

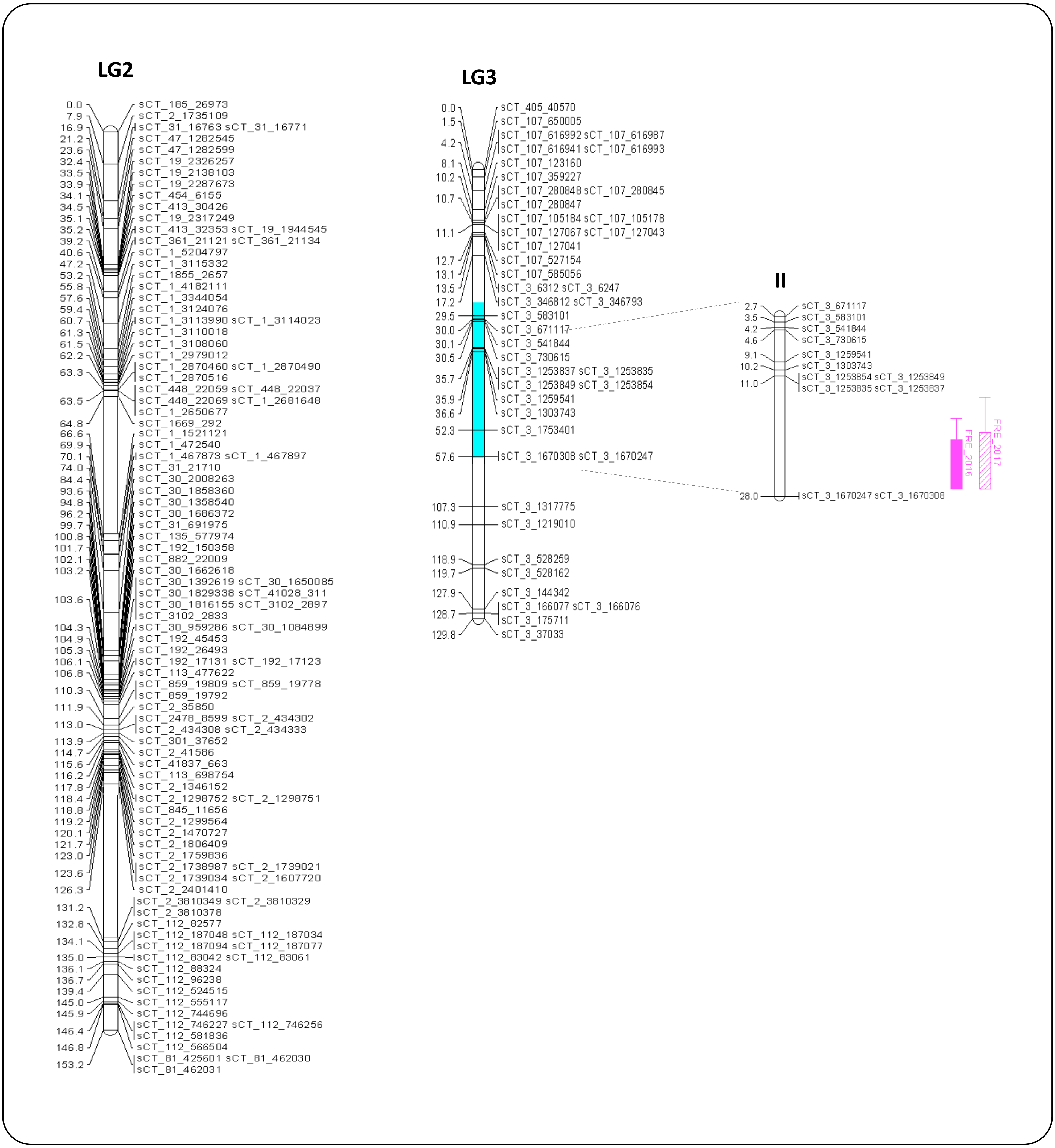

Figure S2 : Genetic map of ‘RB2’ x ‘Sunrise Solo’ and QTL for fruit quality traits (cont.).
The LGs resulted from initial map and final map were labelled by LG1-LG23 and I-X, respectively. The left pane indicates the genetic map position in cM of each SNPs. Homology between both maps was highlighted in turquoise. Colour bars on the right of final map indicate QTL position and LOD interval at 95% confidence; where flesh sweetness (SWE) – red; fruit weight (WEI)-brown; fruit length (LEN)-green; fruit width (WID)-olive; skin freckle (FRE)-pink; flesh thickness (THI)-black; fruit firmness (FIR)- blue. Data from harvest year 2016 and 2017 are represented in solid and diagonal-stripe bar, respectively.

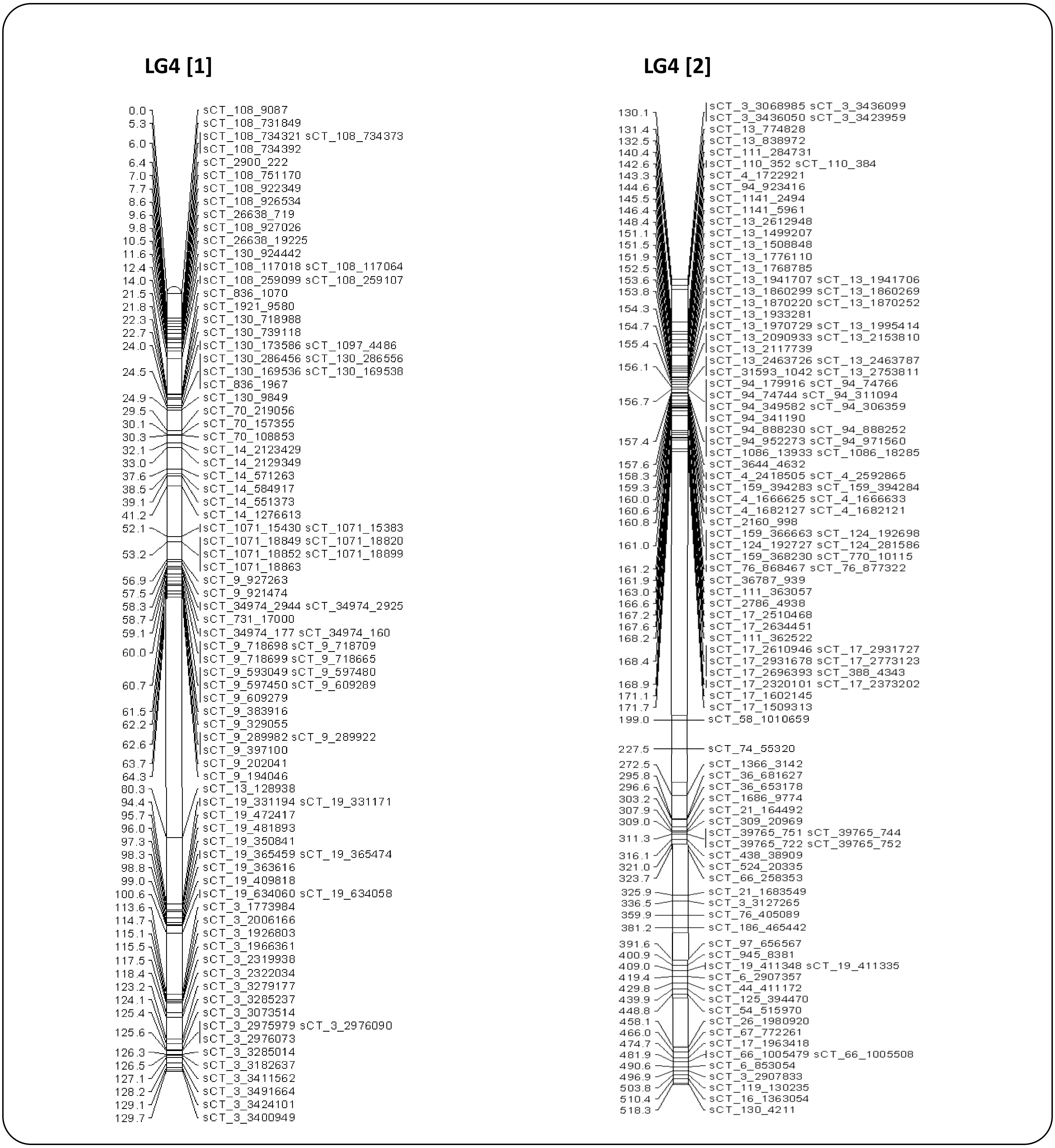

Figure S2 : Genetic map of 'RB2' x 'Sunrise Solo' and QTL for fruit quality traits (cont.).
The LGs resulted from initial map and final map were labelled by LG1-LG23 and I-X, respectively. The left pane indicates the genetic map position in cM of each SNPs. Homology between both maps was highlighted in turquoise. Colour bars on the right of final map indicate QTL position and LOD interval at 95% confidence; where flesh sweetness (SWE) – red; fruit weight (WEI)-brown; fruit length (LEN)-green; fruit width (WID)-olive; skin freckle (FRE)-pink; flesh thickness (THI)-black; fruit firmness (FIR)- blue. Data from harvest year 2016 and 2017 are represented in solid and diagonal-stripe bar, respectively.

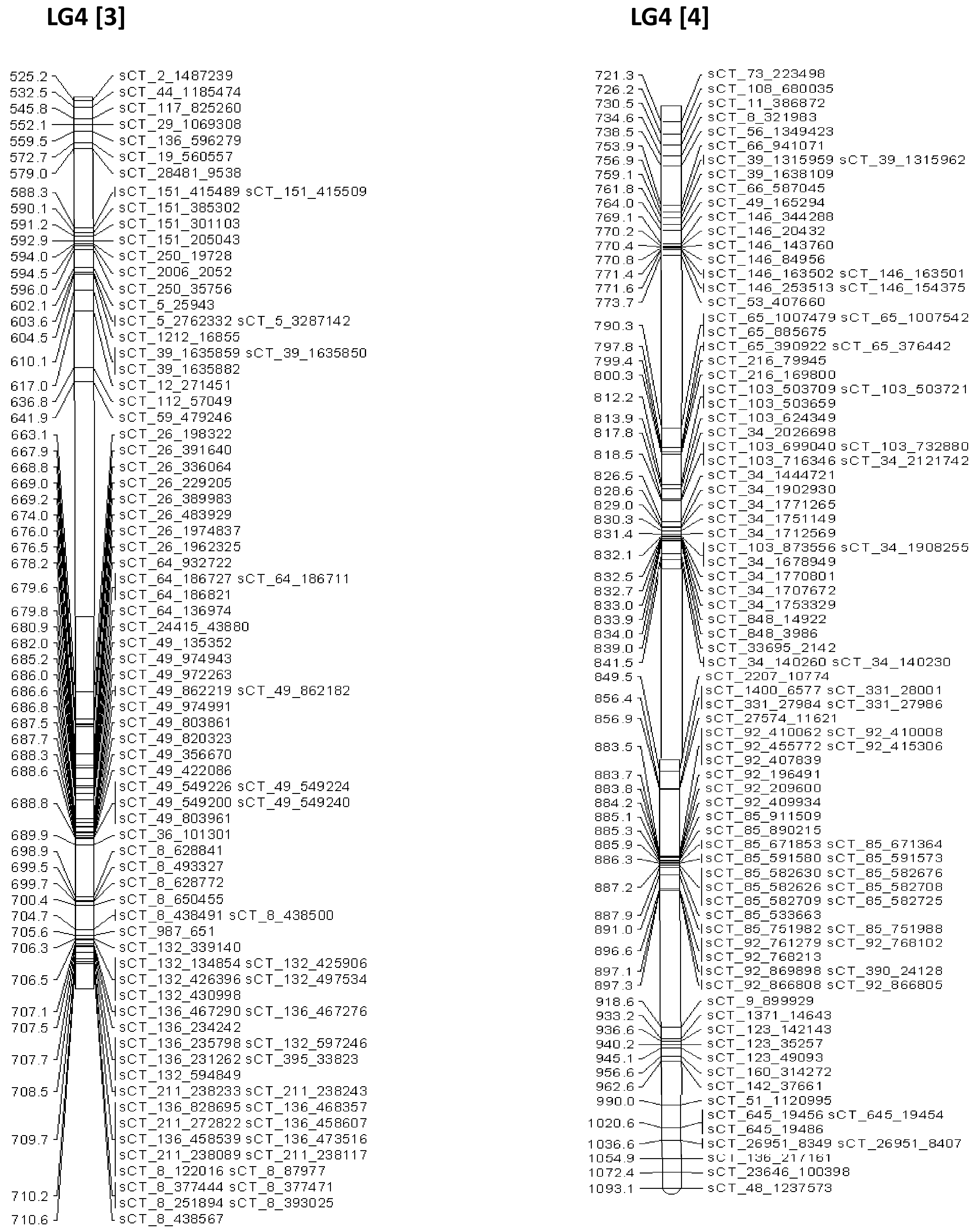

Figure S2 : Genetic map of ‘RB2’ x ‘Sunrise Solo’ and QTL for fruit quality traits (cont.).
The LGs resulted from initial map and final map were labelled by LG1-LG23 and I-X, respectively. The left pane indicates the genetic map position in cM of each SNPs. Homology between both maps was highlighted in turquoise. Colour bars on the right of final map indicate QTL position and LOD interval at 95% confidence; where flesh sweetness (SWE) – red; fruit weight (WEI)-brown; fruit length (LEN)-green; fruit width (WID)-olive; skin freckle (FRE)-pink; flesh thickness (THI)-black; fruit firmness (FIR)- blue. Data from harvest year 2016 and 2017 are represented in solid and diagonal-stripe bar, respectively.

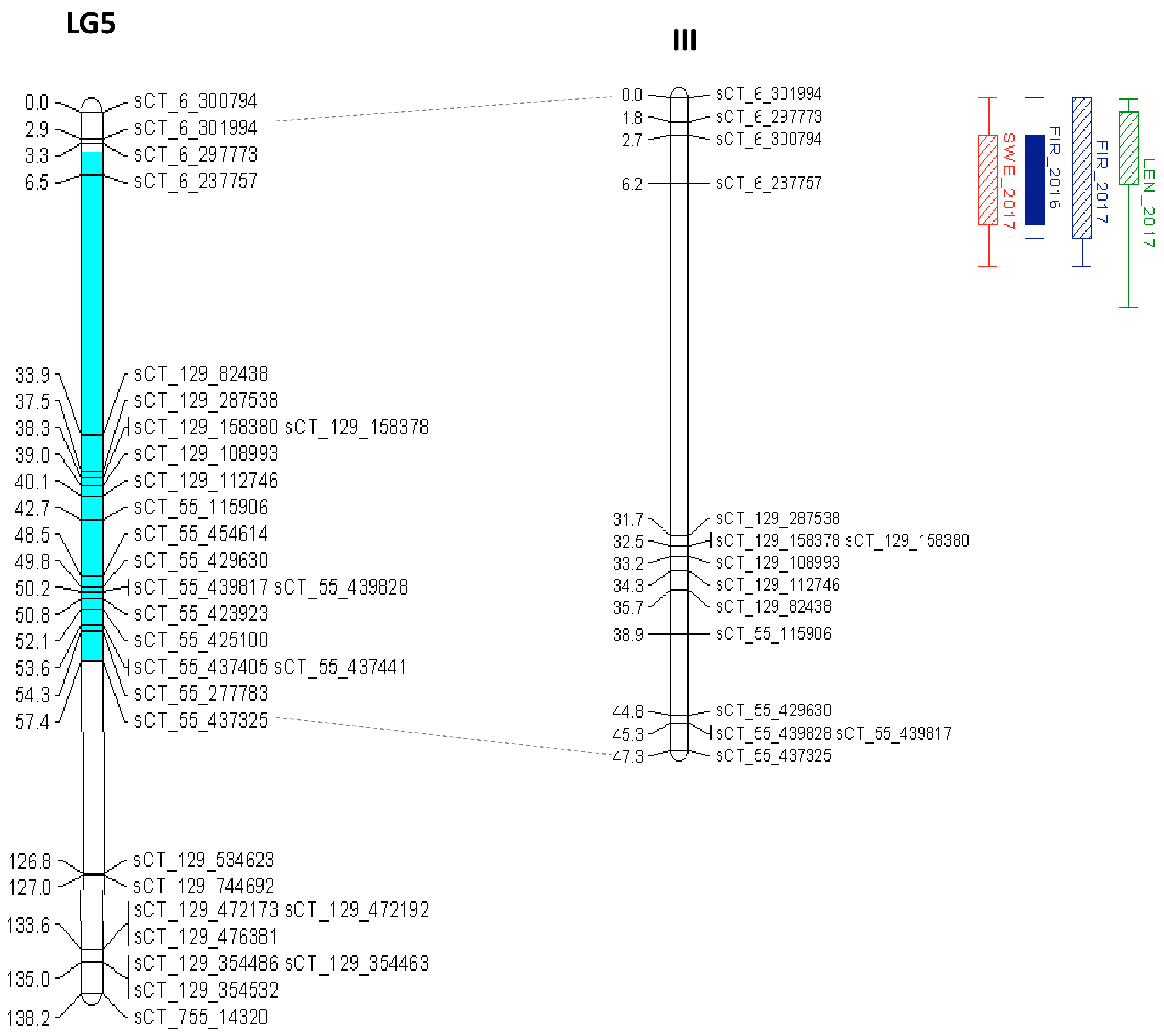

Figure S2 : Genetic map of 'RB2' x 'Sunrise Solo' and QTL for fruit quality traits (cont.).
The LGs resulted from initial map and final map were labelled by LG1-LG23 and I-X, respectively. The left pane indicates the genetic map position in cM of each SNPs. Homology between both maps was highlighted in turquoise. Colour bars on the right of final map indicate QTL position and LOD interval at 95% confidence; where flesh sweetness (SWE) – red; fruit weight (WEI)-brown; fruit length (LEN)-green; fruit width (WID)-olive; skin freckle (FRE)-pink; flesh thickness (THI)-black; fruit firmness (FIR)- blue. Data from harvest year 2016 and 2017 are represented in solid and diagonal-stripe bar, respectively.

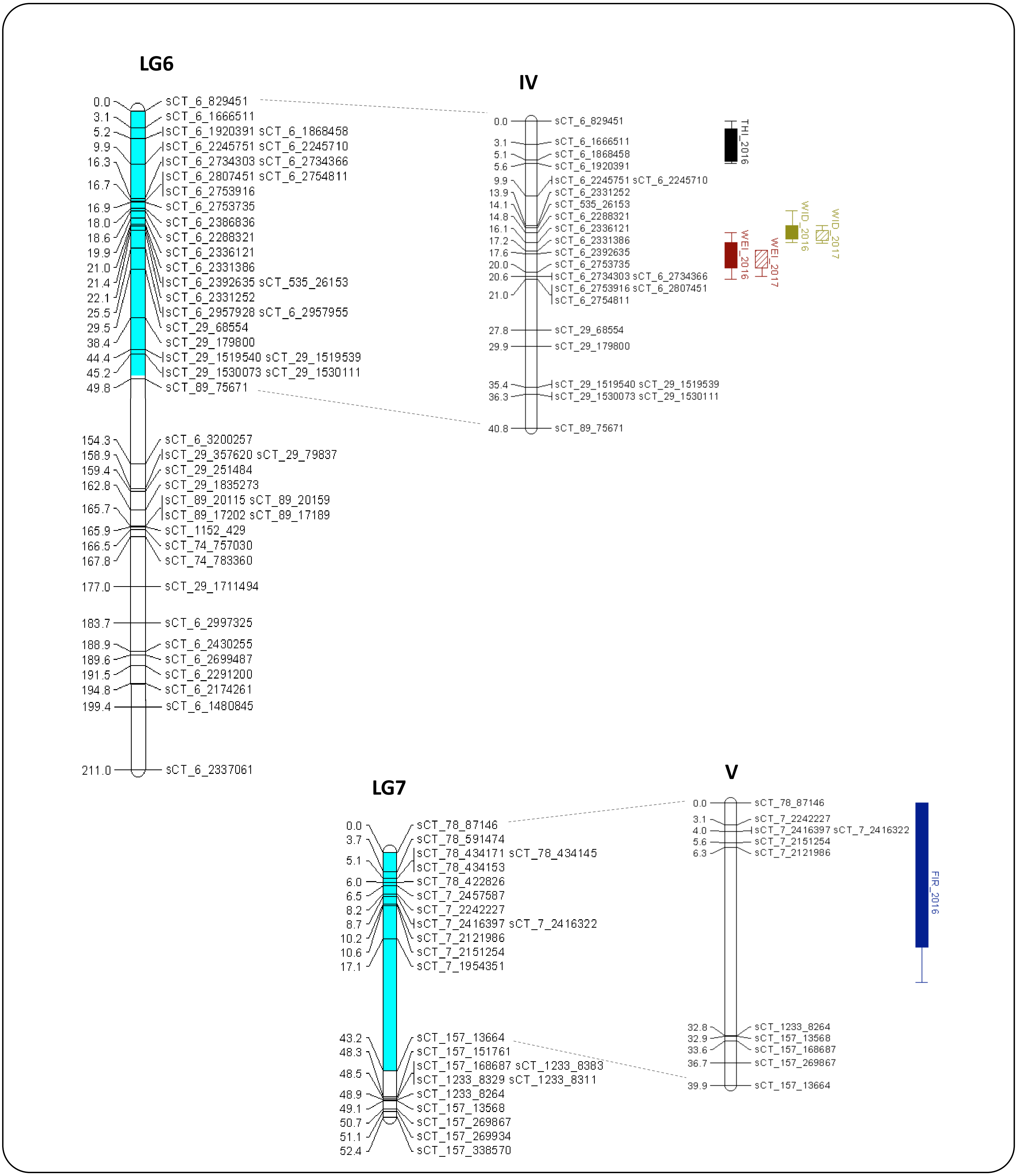

Figure S2 : Genetic map of 'RB2' x 'Sunrise Solo' and QTL for fruit quality traits (cont.).
The LGs resulted from initial map and final map were labelled by LG1-LG23 and I-X, respectively. The left pane indicates the genetic map position in cM of each SNPs. Homology between both maps was highlighted in turquoise. Colour bars on the right of final map indicate QTL position and LOD interval at 95% confidence; where flesh sweetness (SWE) – red; fruit weight (WEI)-brown; fruit length (LEN)-green; fruit width (WID)-olive; skin freckle (FRE)-pink; flesh thickness (THI)-black; fruit firmness (FIR)- blue. Data from harvest year 2016 and 2017 are represented in solid and diagonal-stripe bar, respectively.

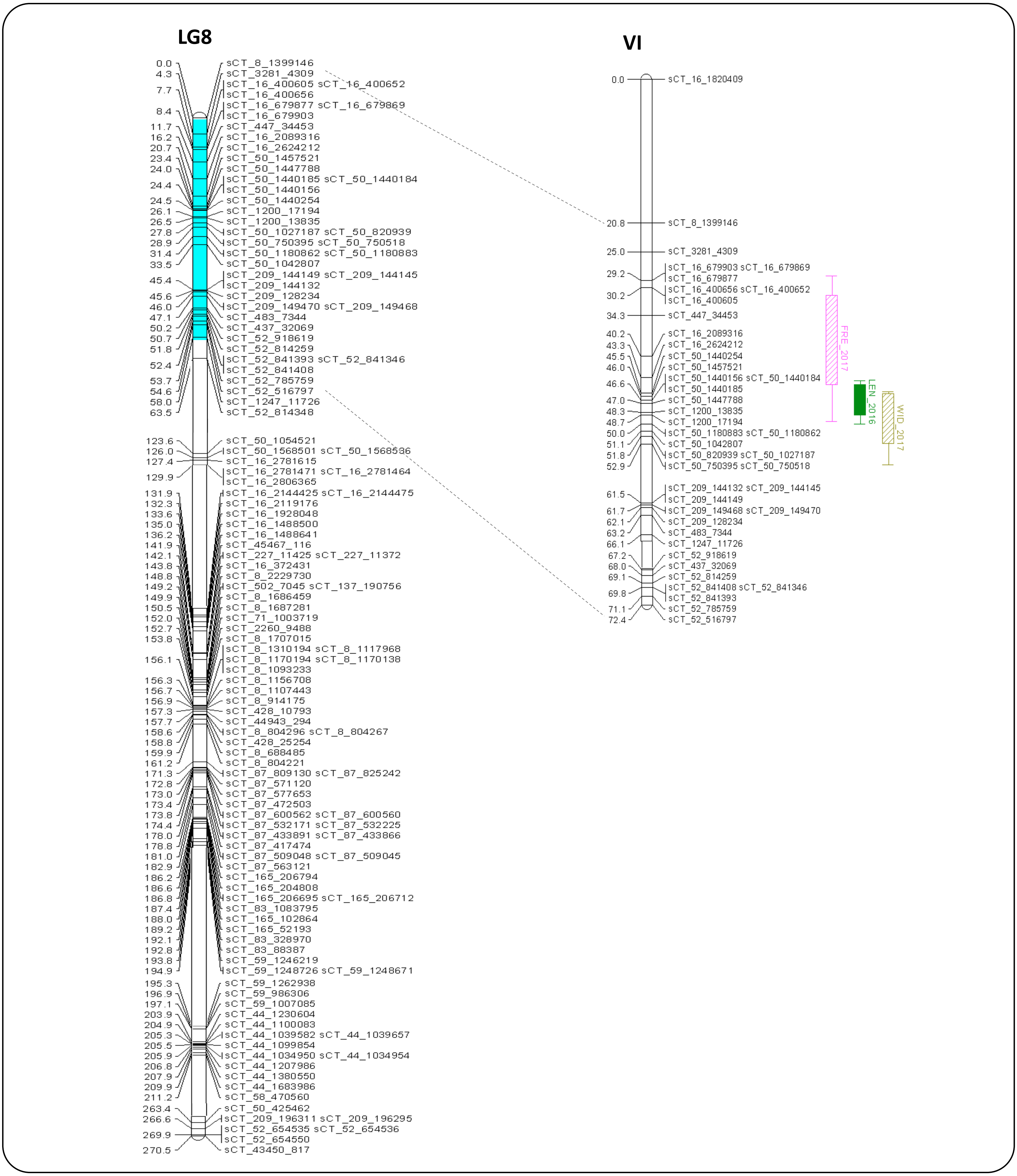

Figure S2 : Genetic map of 'RB2' x 'Sunrise Solo' and QTL for fruit quality traits (cont.).
The LGs resulted from initial map and final map were labelled by LG1-LG23 and I-X, respectively. The left pane indicates the genetic map position in cM of each SNPs. Homology between both maps was highlighted in turquoise ■. Colour bars on the right of final map indicate QTL position and LOD interval at 95% confidence; where flesh sweetness (SWE) – red ■; fruit weight (WEI)-brown ■; fruit length (LEN)-green ■; fruit width (WID)-olive ■; skin freckle (FRE)-pink ■; flesh thickness (THI)-black ■; fruit firmness (FIR)- blue ■. Data from harvest year 2016 and 2017 are represented in solid and diagonal-stripe bar, respectively.

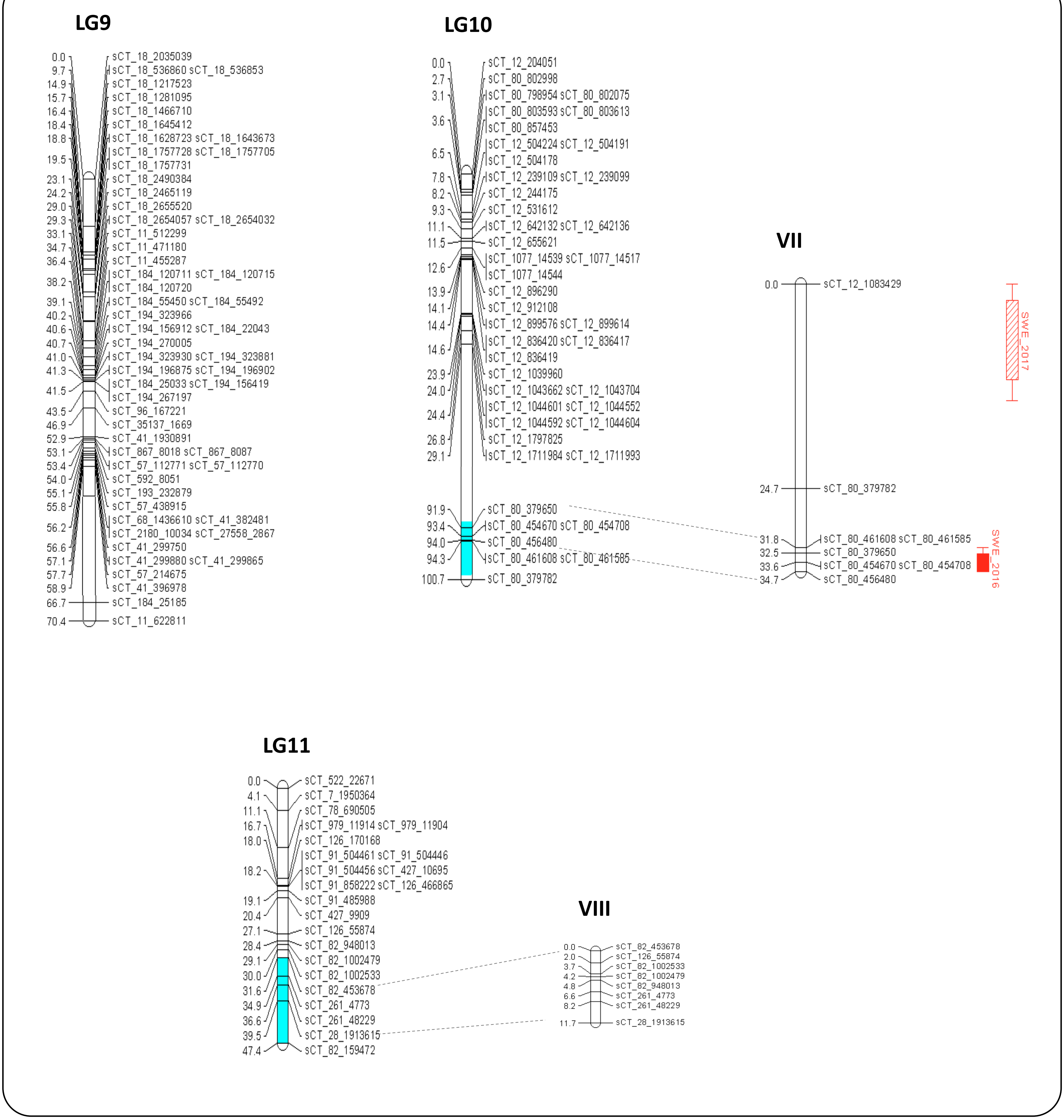

Figure S2 : Genetic map of 'RB2' x 'Sunrise Solo' and QTL for fruit quality traits (cont.).
The LGs resulted from initial map and final map were labelled by LG1-LG23 and I-X, respectively. The left pane indicates the genetic map position in cM of each SNPs. Homology between both maps was highlighted in turquoise. Colour bars on the right of final map indicate QTL position and LOD interval at 95% confidence; where flesh sweetness (SWE) – red; fruit weight (WEI)-brown; fruit length (LEN)-green; fruit width (WID)-olive; skin freckle (FRE)-pink; flesh thickness (THI)-black; fruit firmness (FIR)- blue. Data from harvest year 2016 and 2017 are represented in solid and diagonal-stripe bar, respectively.

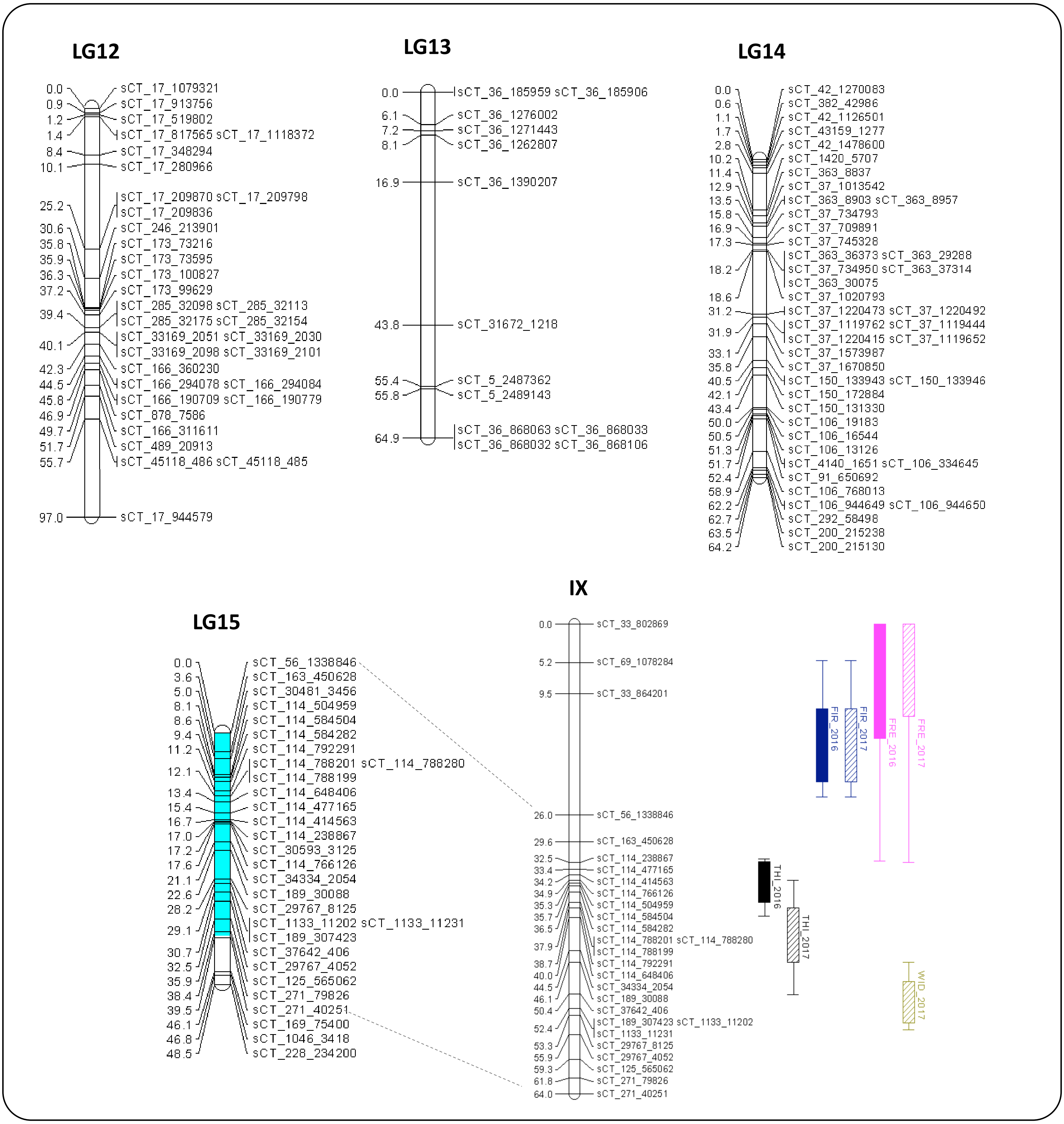

Figure S2 : Genetic map of 'RB2' x 'Sunrise Solo' and QTL for fruit quality traits (cont.).
The LGs resulted from initial map and final map were labelled by LG1-LG23 and I-X, respectively. The left pane indicates the genetic map position in cM of each SNPs. Homology between both maps was highlighted in turquoise. Colour bars on the right of final map indicate QTL position and LOD interval at 95% confidence; where flesh sweetness (SWE) – red; fruit weight (WEI)-brown; fruit length (LEN)-green; fruit width (WID)-olive; skin freckle (FRE)-pink; flesh thickness (THI)-black; fruit firmness (FIR)- blue. Data from harvest year 2016 and 2017 are represented in solid and diagonal-stripe bar, respectively.

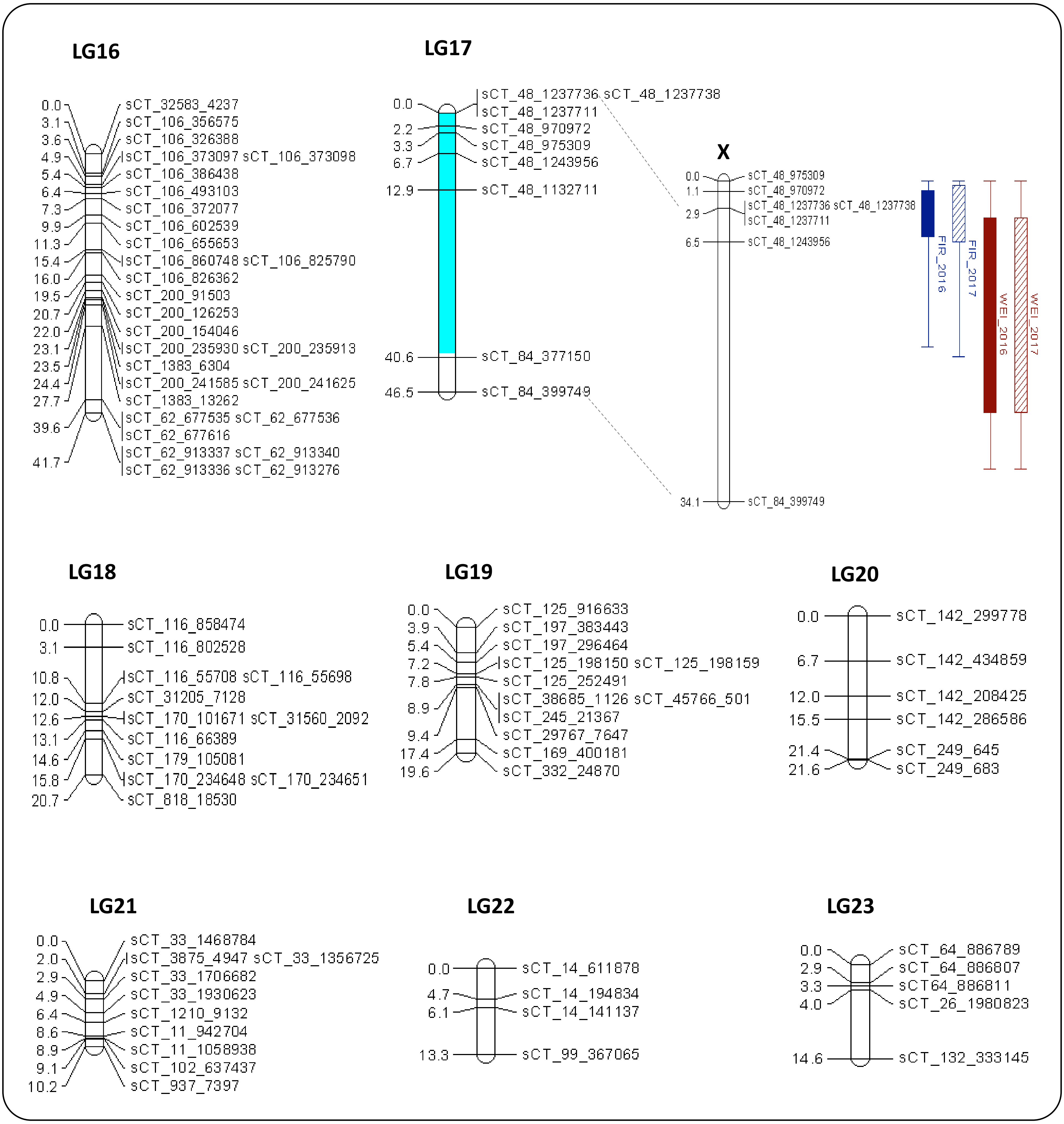

Figure S2 : Genetic map of 'RB2' x 'Sunrise Solo' and QTL for fruit quality traits (cont.).
The LGs resulted from initial map and final map were labelled by LG1-LG23 and I-X, respectively. The left pane indicates the genetic map position in cM of each SNPs. Homology between both maps was highlighted in turquoise . Colour bars on the right of final map indicate QTL position and LOD interval at 95% confidence; where flesh sweetness (SWE) – red ; fruit weight (WEI)-brown ; fruit length (LEN)-green ; fruit width (WID)-olive ; skin freckle (FRE)-pink ; flesh thickness (THI)-black ; fruit firmness (FIR)- blue . Data from harvest year 2016 and 2017 are represented in solid and diagonal-stripe bar, respectively.
