# Supplementary material for: Linkage mapping and quantitative trait loci analysis of sweetness and other fruit quality traits in papaya
Source: BMC Plant Biol. 2019 Oct 26;19:449. doi: 10.1186/s12870-019-2043-0 (PMC6815024; doi:10.1186/s12870-019-2043-0)
Supplement: Supplementary file 7 — Additional file 7: Table S5. Associated SNPs and candidate genes for flesh sweetness and other fruit quality traits. [file 12870_2019_2043_MOESM7_ESM.pdf]

Table S5: Associated SNPs and candidate genes for flesh sweetness and other fruit quality traits

Trait	Marker	Candidate genes	Gene functional annotation	Candidate genes GO terms	Corresponding gene function
Flesh sweetness	sCT_80_454708	CP00080G00550	Non-canonical poly(A) RNA polymerase PAPD5	GO:0016779, GO:0051301; GO:0071044	Nucleotidyltransferase activity, cell division, Histone mRNA catabolic process
	sCT_80_454708	CP00080G00540	KIN17-like protein	GO:0006260; GO:0006974; GO:0048638	DNA replication; cellular response to DNA damage stimulus, regulation of developmental growth
	sCT_12_1083429	CP00012G01230	Transmembrane emp24 domain-containing protein P24-beta2	GO:0016192, GO:0006886, GO:0008320	Vesicle-mediated transport; intracellular protein transport, protein transmembrane transporter activity
Fruit weight	sCT_20_560108	CP00020G00540	Protein trichome birefringence-like 12	GO:0071554	Cell wall organization or biogenesis
	sCT_6_2754743	CP00006G03490	Serine/threonine-protein kinase/endoribonuclease IRE1	GO:0004674	Protein serine/threonine kinase activity
	sCT_6_2754743	CP00006G03500	Glutamate receptor 3	GO:0008066	Glutamate receptor activity
	sCT_20_544712	CP00020G00510	Fatty acid amide hydrolase-like	GO:0017064; GO:0009505; GO:0004128	Fatty acid amide hydrolase activity; plant-type cell wall; cytochrome-b5 reductase activity, acting on NAD(P)H
	sCT_6_2392635	CP00006G03260	IST1-like protein	GO:0015031	Protein transport
Fruit length	sCT_50_1447788	CP00050G01530	Exopolygalacturonase-like	GO:0016021; GO:0005975	Integral component of membrane; carbohydrate metabolic process
	sCT_42_954411	CP00042G00970	NAC domain-containing protein 41-like	GO:0005975	Carbohydrate metabolic process
	sCT_42_954411	CP00042G00960	*Carica papaya* chromosome Y sequence		
	sCT_42_954411	CP00042G00980	Prolyl 4-hydroxylase 9	GO:0019538	Protein metabolic process
Fruit width	sCT_6_2331252	CP00006G03140	Bifunctional nuclease 2	GO:0032296	Double-stranded RNA-specific ribonuclease activity
Skin freckle	sCT_33_864201	CP00033G00820	Ultraviolet-B receptor UVR8	GO:0009881, GO:0006281	Photoreceptor activity, DNA repair
	sCT_16_2143939	CP00016G01460	Putative disease resistance protein RGA1	GO:0006952, GO:0009723, GO:0009737	Defence response, response to ethylene, abscisic acid
Fruit firmness	sCT_6_237757	CP00006G00370	UPF0553 protein-like	GO:0005575; GO:0006400	Cellular component; tRNA modification
	sCT_7_2121986	CP00007G02090	DNA-directed RNA polymerase III subunit	GO:0001056	RNA polymerase III activity
	sCT_48_1243956	CP00048G02250	MYB-like protein X	GO:0003677; GO:0001135	DNA binding; transcription factor activity, RNA polymerase II transcription factor
	sCT_48_1243956	CP00048G02260	Pectin acetyl esterase 12-like	GO:0045490	Pectin catabolic process
Flesh thickness	sCT_6_1666511	CP00006G02040	Cytochrome P450 84A1-like	GO:0009809, GO:0016020	Lignin biosynthetic process, membrane,
	sCT_6_1666511	CP00006G02050	Protein FMP32, mitochondrial	GO:0033617	Mitochondrial respiratory chain complex IV assembly
	sCT_114_766126	CP00114G00550	Ethylene-responsive transcription factor RAP2-7-like	GO:0009873	Ethylene-activated signalling pathway
